# Supplementary material for: Impact of BCG revaccination on the response to unrelated vaccines in a Ugandan adolescent birth cohort: randomised controlled trial protocol C for the ‘POPulation differences in VACcine responses’ (POPVAC) programme
Source: BMJ Open. 2021 Feb 16;11(2):e040430. doi: 10.1136/bmjopen-2020-040430 (PMC7893605; doi:10.1136/bmjopen-2020-040430)
Supplement: Supplementary data [file bmjopen-2020-040430supp001.pdf]

SUPPLEMENTARY INFORMATION

The impact of Bacillus Calmette-Guérin revaccination on the response to unrelated vaccines in a Ugandan adolescent birth cohort: randomised controlled trial protocol C for the 'POPulation differences in VACcine responses' (POPVAC) programme

Ludoviko Zirimenya[1,¶], Gyaviira Nkurunungi[1,¶,*], Jacent Nassuuna[1,¶], Agnes Natukunda[1,¶], Alex Mutebe[1], Gloria Oduru[1], Grace Kabami[1], Hellen Akurut[1], Caroline Onen[1], Milly Namutebi[1], Joel Serubanja[1], Esther Nakazibwe[1], Florence Akello[1], Josephine Tumusiime[1], Moses Sewankambo[1], Samuel Kiwanuka[1], Fred Kiwudhu[1], Robert Kizindo[1], Anne Wajja[1], Stephen Cose[1,2], Moses Muwanga[3], Emily L Webb[4], Alison M Elliott[1,2] **for the POPVAC trial team**

[1]Immunomodulation and Vaccines Programme, Medical Research Council/Uganda Virus Research Institute and London School of Hygiene and Tropical Medicine (MRC/UVRI and LSHTM) Uganda Research Unit, Entebbe, Uganda

[2]Department of Clinical Research, London School of Hygiene and Tropical Medicine, London, United Kingdom

[3]Entebbe Hospital, Entebbe, Uganda

[4]MRC Tropical Epidemiology Group, Department of Infectious Disease Epidemiology, London School of Hygiene and Tropical Medicine, London, United Kingdom

[¶]These authors contributed equally

***Correspondence:** Gyaviira Nkurunungi; Gyaviira.Nkurunungi@mrcuganda.org

Zirimenya L, *et al. BMJ Open* 2021; 11:e040430. doi: 10.1136/bmjopen-2020-040430

Table S1: Schedule of visits and procedures

**VISIT NUMBER**	**1**	**2**	**3**	**3.2, 3.3**	**4**	**5**	**6**
**WEEKS FROM 1ST IMMUNISATION**	**-4 to 0[1]**	**0**	**4**	**4 weeks +4 days**	**8**	**28**	**52 SE**
	**Screening**	**Immunisation**	**Immunisations**		**Primary endpoint (PE)**	**Immunisations**	**Secondary endpoint (SE)**
**RANDOMISED BCG "IMMUNISATION"**							
BCG arm (x)		**x**					
No BCG arm (o)		**o**					
**ANTHELMINTHIC TREATMENT**							
Praziquantel and albendazole or mebendazole					**X[2]**	**X[2]**	**X[2]**
**VACCINES**							
YF-17D			x				
Ty21a			X[6]				
HPV			x		[x][4]	x	
Td						x	[x][5]
**INVESTIGATIONS/PROCEDURES**							
Inclusion/exclusion criteria	x						
Informed consent	x						
Questionnaire	x		x	x	x	x	x
Examination	x		(x)	(x)	(x)	(x)	(x)
Urine β-HCG test (female only) 1mL	x	X[5]	x			x	
Urine YF viral load				x			
Stool for PCR and storage	x						x
Stool for coproantibody and storage	x				x		
**BLOOD TESTS**							
Malaria PCR (1ml)	x						x
Serology for HIV, prior malaria and *S. mansoni* (0.5 ml)	x						
Mansonella perstans (1ml)	x						
Full blood count (1ml)	x		x				
Assessments of pre-immunisation responses, and/or vaccine response outcomes and/or exploratory immunology; storage[9] (10-20ml)	x		x		x		x
Blood for gene expression (2ml)	x		x				
Blood vol (mL)	27		17		20		25
Cumulative blood vol (mL)[8]	27		44		64		89

PE: primary endpoint; **SE:** secondary endpoint
Immunisation days highlighted in green, primary end point days in red
(**x**) performed if clinically indicated
1. Screening and enrolment into Project C will take place shortly before enrolment, sometimes on the same day

2. Treatments given after sample when schedules coincide
3. Week 8 HPV dose will be given for previously-unvaccinated girls aged ≥14 years
4. Week 52 Td booster dose will be provided as a service
5. Pregnancy test to be repeated if more than 4 weeks elapses between screening and immunisation
6. Oral typhoid vaccine doses will be administered on three alternate days namely visit 3, 3.1, and 3.2
7. Exploratory immunology blood volume will be guided by guidelines from Harvard Mass General, where a maximum of 3ml/kg body weight is taken at any one time point and not more than 3ml/kg is taken over any 8-week period (ref http://www.drgreene.com/21_1616.html.) These guidelines have been followed in a previous study vaccinating adolescents with investigational tuberculosis vaccine MVA85A (in Uganda).[1] The total blood volume planned is 64 ml over the initial intensive sampling period of 8 weeks. Revision of sample volumes based on weight will only be required for participants who weigh less than 21 kg; the average weight of children aged 9 years is expected to be 28kg (with 21kg the 3rd centile) with greater weights for older children.[2]

23

Further rationale for the selection of vaccines

Yellow fever vaccine

Yellow fever vaccine YF-17D is a live replicating parenteral vaccine. The vaccine (Stamaril; Sanofi Pasteur) is available for purchase in Uganda. Yellow Fever (YF) causes outbreaks in Uganda and the wider region[3] and YF-17D is a candidate for Uganda's expanded programme on immunisation (EPI; H Luzze, personal communication). As noted above, lower vaccine replication, lower neutralising antibody induction, and greater waning, are described in Uganda compared to Switzerland.[4] YF-17D is a potential vector for novel vaccine constructs,[5] adding relevance to vaccine development.

Typhoid vaccine Ty21a

Typhoid vaccine Ty21a is a live replicating oral vaccine and also a potential vector for new vaccine constructs.[6] Ty21a vaccine will be purchased from PaxVax, Redwood City, California. Substantial, multi-year typhoid outbreaks occur in Uganda and immunisation campaigns have been advocated as cost effective.[7]

Ty21a was developed in the 1970s. Although not routinely used in Uganda, it has been (and is currently) registered in many countries. It was first registered in the United States and United Kingdom in the 1980s, and is recommended by the World Health Organisation for both endemic and epidemic settings.[8] It has comparable efficacy to the parenteral Vi polysaccharide typhoid vaccine, good durability and minimal adverse effects.[8] It is proposed for use in this study to model effects of study exposures and intervention on the response to a live oral vaccine.

The Ty21a vaccine is given as a three-dose regimen on alternate days.

Human Papilloma Virus (HPV) vaccine

Human Papilloma Virus (HPV) vaccine is a protein virus-like particle. The quadrivalent HPV Vaccine Gardasil (Merck) is available for purchase in Uganda and is the vaccine used by the national EPI programme. HPV immunisation is being rolled out among girls to prevent cervical neoplasia, the commonest cancer among Ugandan women and we will coordinate provision with the national HPV immunisation programme.[9] HPV immunisation is also beneficial for boys since HPV infection is associated with anogenital warts, anal cancer and oropharyngeal cancers in both males and females, and with penile cancer in men,[10] and we will include boys in these studies.

Tetanus and diphtheria vaccines

Tetanus and diphtheria vaccines comprise inert toxoids (Td). Booster immunisation is recommended for young women to prevent maternal and neonatal tetanus. Recent evidence emphasises the need to protect young men also.[11]

Immunisation Postponement Criteria

If any one of the following is identified at the time scheduled for immunisation, the participant may be immunised at a later date, or withdrawn, at the discretion of the Investigator. The participant must be followed until resolution of the event as with any adverse event:

- Acute disease at the time of immunisation. Acute disease is defined as the presence of a moderate or severe illness with or without fever. All vaccines can be administered to persons with a minor illness such as diarrhoea or mild upper respiratory infection with or without low-grade fever, i.e. temperature of ≤37.5°C (99.5°F)
- Temperature of >37.5°C (99.5°F) at the time of immunisation
- Taking antibiotics or antimalarials currently, or within the past 7 days, of the date of Ty21a administration (ascertained verbally)

Vaccine storage and transport

In order to maintain a reliable vaccine cold chain, the vaccines and diluents to be used will be stored and transported within the recommended temperature range of +2°C to +8°C. Care will be taken to ensure that the vaccines are not frozen. BCG, being sensitive to light, will be kept in the dark (normally within its secondary packaging) for as long as possible to protect it during storage and transportation. All vaccines will be kept in appropriate refrigeration equipment with a temperature monitoring device to ensure temperatures remain between +2°C and +8°C. Cold boxes/vaccines carriers with temperature monitors will be used to transport vaccines and the diluents from the MRC/UVRI and LSHTM Uganda Research Unit (Entebbe) to the clinic where vaccination will take place and while transporting vaccines to immunisation sessions. Designated staff will be given responsibility for managing the vaccine cold chain. All cold chain equipment including the temperature monitoring devices used for this project will comply with relevant technical specifications as defined by the EPI standards. Basic routine maintenance will be regularly carried out on all cold chain equipment.

Additional laboratory measurements

Additional assays will comprise measurement of parasite infection exposure, HIV serology, pregnancy testing and full blood counts. HIV testing and pregnancy testing will be accompanied by appropriate counselling by trained staff.

Current *S. mansoni* infection status and intensity will be determined by serum/plasma levels of circulating anodic antigen (CAA). The method is quantitative, highly specific for *Schistosoma* infection, and much more sensitive than the conventional Kato Katz method.[12] CAA will be assessed retrospectively on stored samples collected at baseline.

Zirimenya L, *et al. BMJ Open* 2021; 11:e040430. doi: 10.1136/bmjopen-2020-040430

Prior exposure to schistosomiasis will be evaluated by ELISA for IgG to schistosome egg antigen using stored blood samples collected at baseline.

The presence of other helminth infections will be determined retrospectively using stool PCR of samples collected at baseline and at weeks 28 and 52.[13] In accordance with national guidelines, all participants will be treated with albendazole or mebendazole after collection of samples for primary endpoints at week 8 and 28, and after collection of samples for secondary endpoints at week 52.

Current malaria infection status and intensity will be assessed retrospectively by PCR on stored samples collected on immunisation days and at week 52.

Malarial fever: Individuals presenting with fever will be investigated using rapid diagnostic tests for malaria and treated based on the results and according to prevailing national guidelines.

Prior malaria exposure will be evaluated by ELISA for IgG to malaria antigen using stored samples collected at baseline.

HIV serology will be done on blood samples using rapid tests and according to prevailing national algorithms. The current algorithm is shown in Appendix 2. This will be done at baseline.

Pregnancy testing will be done using urine samples and standard operating procedures for assessment of urine [illegible]-human chorionic gonadotropin[illegible]hCG). This will be done at baseline and before immunisation on each immunisation day.

Full blood counts will be conducted using a haematology analyser. Mild, moderate and severe anaemia will be defined according to WHO guidelines, by age.[14] This will be done at baseline (to test for anaemia as part of the eligibility assessment), and pre-immunisation as part of the assessment of immunological profile.

Individuals found to be HIV positive or pregnant will be referred to appropriate providers for further care.

Individuals with severe anaemia (haemoglobin <82g/L) will be excluded from the randomised intervention (since the intervention might be beneficial in management of anaemia). They will be treated for anaemia and for any underlying cause identified.

Operational considerations

Programme governance

A Programme Steering Committee will be set up to guide progress across all projects. This will comprise the following:

- An independent chair
- Representatives from the Ministry of Health programmes for immunisation and for vector borne disease control
- Representatives of district authorities (Mukono and Jinja districts)
- Community representatives
- Principal investigator and co-investigators
- Project leader and post-doctoral immunologist
- Trial statistician
- Laboratory manager
- Medical Research Council observer

Informed consent

Both written informed assent from the participants and written informed consent from a parent or guardian will be required for participation, although these may not necessarily be obtained at the same time. Information will be provided in both English and the appropriate local language. For individuals who cannot speak the languages used, or who cannot read or write, a witness who can read the information sheet and translate the information to the participant or parent/guardian will be used. Informed consent by emancipated or mature minors will be obtained using designated consent form for these kinds of participants.

The aims of the study, all tests, treatments and immunisations to be carried out and potential risks will be explained. The participant will be given the opportunity to ask about details of the trial, and will then have time to consider whether or not to participate. If they do decide to participate, they and their parent/guardian will sign and date two copies of the assent and consent forms, one for them to take away and keep, and one to be stored securely by the research team. Separate information and consent forms will be provided for consent for storage of samples for future studies and for anonymous sharing of data from this study. For the EMaBS cohort genetic data are already available based on previous approval; the information sheet will explain that these data may be used in analyses related to this protocol.

Screening and Eligibility Assessment

Once the informed consent process has been completed, and consent (and assent) given, a baseline medical history (including concomitant medication) will be collected. Vital signs will be checked and a physical examination will be performed. Inclusion and exclusion criteria will be checked.

Participants will undergo pre- and post-test counselling for HIV and (for girls) pregnancy testing by a trained and experienced nurse- or clinician-counsellor. Blood, urine and stool samples will be obtained, for tests as specified in the schedule of procedures (Appendices A-C). These tests are to exclude the major, immunomodulating co-infection, HIV, and conditions that might impact safety (anaemia, pregnancy).

Enrolment

Participants who consent/assent, complete the screening processes, satisfy all the inclusion criteria and meet none of the exclusion criteria will be enrolled into the trial. On the enrolment day (which may be the same as the screening day in some cases) eligibility will be checked and participants will be enrolled sequentially to the next randomisation number. They will then be given BCG vaccine or not, according to their allocation.

Discontinuation / withdrawal criteria

In accordance with the principles of the current revision of the Declaration of Helsinki and any other applicable regulations, a participant has the right to withdraw from the study at any time and for any reason, and is not obliged to give his or her reasons for doing so. The Investigator may withdraw the participant at any time in the interests of the participant's health and well-being. In addition, the participant may withdraw/be withdrawn for any of the following reasons:

- Ineligibility (either arising during the study or retrospectively, having been overlooked at screening)
- Administrative decision by the Investigator
- Significant protocol deviation
- Participant non-compliance with study requirements
- An adverse event which requires discontinuation of the study involvement or results in inability to continue to comply with study procedures.

Any participant who becomes pregnant during the trial will be followed up until the end of the pregnancy but no further immunisations will be given unless indicated during pregnancy (as is the case for tetanus toxoid). The trial allocation for this participant will be unblinded and the participant will only be given further treatment if clinically indicated. The babies will also be followed up and examined for any adverse effects. We will not routinely perform venepuncture in a pregnant participant.

The reason for withdrawal will be recorded in the case report form (CRF). If withdrawal is due to an AE, appropriate follow-up visits or medical care will be arranged, with the agreement of the participant, until the AE has resolved, stabilised or a non-trial related causality has been assigned.

If a participant withdraws from the study samples collected before their withdrawal from the trial will be used/ stored unless the participant specifically requests otherwise.

Trial discontinuation

The Trial will be discontinued in the event of new scientific information that renders continuation futile or unethical, or for any other reason, at the discretion of the Programme Steering Committee.

End of study definition

The trial will be completed when the last participant enrolled into the trial has completed their final follow up visit.

Safety assessments and oversight

No new investigational drug or product will be used in the proposed trial. However, standard approaches for monitoring safety and reporting of serious adverse events will be followed.

Monitoring

The trial will be monitored by both internal and external monitors according to a pre-defined monitoring plan which will include a site initiation visit, monitoring visits at least annually, and a close-out visit. The monitors will assess patient safety, data integrity, and adherence to the protocol and to Good Clinical Research Practice procedures.

Procedures to be followed in the event of abnormal findings

Abnormal clinical findings from medical history, examination or blood tests will be assessed as to their clinical significance throughout the trials. If an abnormal test result is deemed clinically significant, it may be repeated. If a test remains clinically significant, the participant will be informed and appropriate medical care arranged as appropriate and with the permission of the participant. Specific details regarding findings, discussion with participants and resulting actions will be recorded in the clinical records. Decisions to exclude the participant from enrolling in the trial or to withdraw a participant from the trial will be at the discretion of the Investigator.

Zirimenya L, *et al. BMJ Open* 2021; 11:e040430. doi: 10.1136/bmjopen-2020-040430

Data and Safety Monitoring Board (DSMB)

A data and safety monitoring board (DSMB) will be appointed to provide real-time safety oversight. The DSMB will be notified within 7 days of the Investigators' being aware of the occurrence of SAEs. The DSMB may recommend the Investigators to place the trial on hold if deemed necessary following an intervention-related SAE. The DSMB will be chaired by a clinician experienced in clinical trials. There will be a minimum of two other appropriately qualified committee members. In the case of events related to a blinded intervention, the DSMB can request unblinding. Membership will include a statistician, and at least one Ugandan member. All correspondence between Investigators and the DSMB will be conveyed by the Principal Investigator to the trial Sponsor. The Chair of the DSMB will be contacted for advice and independent review by the Investigator or trial Sponsor in the following situations:

- The occurrence of any SAE
- Any other situation where the Investigator or trial Sponsor feels independent advice or review is important.

Ethical and regulatory considerations

Information regarding risks and benefits to the participant

Participants in this programme will be adolescents and therefore a vulnerable human population. Care will be taken to provide adequate, age and education-status appropriate information and to ensure that it is understood; and to emphasise that participation is voluntary. Participants will be enrolled only when they have given their own assent and when consent has been given by the parent or guardian.

No major risks to the participants are anticipated since all the treatments and vaccines to be given are licensed and known to be safe. The main risk to participants will be time lost from school work: we will work with parents to minimise disruption to studies.

Participants will suffer the discomfort and inconvenience of providing blood samples (and stool and urine samples). Occasionally people faint when a vaccine is given or when blood is drawn. Individuals will be comfortably seated during these procedures and the research team will be trained to manage such events.

The immunisations to be given have recognised side effects which are usually mild and resolve spontaneously in a few days to one week. Parenteral vaccines are likely to result in pain and swelling at the site of injection and mild fever; very occasionally pain and swelling can be severe and associated with difficulty in moving the shoulder. Sometimes headache and tiredness occurs. Rarely

a vaccine may cause a severe allergic reaction. For most vaccines this is estimated at less than one in a million doses (but 1 in 55,000 for Yellow Fever vaccine).[15] Individuals with a history of a possible allergic reaction to drugs or vaccines, or to vaccine components including eggs or chicken proteins, will be excluded from the studies. The research team will be trained and prepared to manage severe allergic reactions.

Adverse reactions to Yellow Fever vaccine include severe nervous system reaction (about 1 person in 125,000) and severe, life-threatening illness with organ failure (about 1 person in 250,000). The mortality for this severe, life-threatening adverse effect is reported as about 50%.[15]

BCG immunisation is likely to induce a scar in many cases. This may develop over several weeks, starting as a small papule at the injection site which may become ulcerated and then heal over a period of 2 to 5 months; and lymphadenopathy may develop. Occasionally a more severe local reaction occurs (estimated at 1 per 1,000-10,000 doses): for example, an abscess develops and scars may develop into keloids. Rarely BCG can cause disseminated disease (1 per 230,000 to 640,000 doses), or disease in sites remote from the immunisation site. Disseminated BCG disease usually occurs in immunocompromised people: HIV positive people will be excluded from these studies.[16] BCG "pre-immunisation" may interfere with the response to the subsequent live vaccines; indeed our hypothesis, and published results, suggest that it may suppress replication of YF 17D vaccine.[17] However, this reduced replication has not been shown to correlate with, or result in, reduced levels of neutralising antibody titres (which are the desired protective outcome).[4,17]

Oral typhoid vaccine (Ty21a) may occasionally be associated with stomach pain, nausea, vomiting and (rarely) rash.[15]

Benefits

All the vaccines to be given are licensed and regarded as safe. In general, the vaccines and treatments are expected to provide protection against infectious diseases. Participants and their families, and communities are expected to benefit from improved understanding of vaccines.

BMJ Publishing Group Limited (BMJ) disclaims all liability and responsibility arising from any reliance placed on this supplemental material which has been supplied by the author(s)

References

1. Wajja A, Kizito D, Nassanga B, et al. The effect of current Schistosoma mansoni infection on the immunogenicity of a candidate TB vaccine, MVA85A, in BCG-vaccinated adolescents: An open-label trial. *PLoS Negl Trop Dis* 2017;11(5):e0005440. doi: 10.1371/journal.pntd.0005440 [published Online First: 2017/05/05]

2. WHO. Growth reference 5-19 years. 2007

3. Kraemer MU, Faria NR, Reiner RC, Jr., et al. Spread of yellow fever virus outbreak in Angola and the Democratic Republic of the Congo 2015-16: a modelling study. *The Lancet Infectious diseases* 2016 doi: 10.1016/s1473-3099(16)30513-8 [published Online First: 2016/12/27]

4. Muyanja E, Ssemaganda A, Ngauv P, et al. Immune activation alters cellular and humoral responses to yellow fever 17D vaccine. *The Journal of clinical investigation* 2014;124(7):3147-58. doi: 10.1172/jci75429 [published Online First: 2014/06/10]

5. Aguiar M, Stollenwerk N, Halstead SB. The Impact of the Newly Licensed Dengue Vaccine in Endemic Countries. *PLoS neglected tropical diseases* 2016;10(12):e0005179. doi: 10.1371/journal.pntd.0005179 [published Online First: 2016/12/22]

6. Dharmasena MN, Osorio M, Filipova S, et al. Stable expression of Shigella dysenteriae serotype 1 O-antigen genes integrated into the chromosome of live Salmonella oral vaccine vector Ty21a. *Pathogens and disease* 2016 doi: 10.1093/femspd/ftw098 [published Online First: 2016/09/23]

7. Carias C, Walters MS, Wefula E, et al. Economic evaluation of typhoid vaccination in a prolonged typhoid outbreak setting: the case of Kasese district in Uganda. *Vaccine* 2015;33(17):2079-85. doi: 10.1016/j.vaccine.2015.02.027 [published Online First: 2015/02/26]

8. WHO. Position Paper on Typhoid vaccines: WHO position paper – March 2018 2018

9. Centre HI. HPV and related diseases report: Uganda. 2016. http://www.hpvcentre.net/statistics/reports/UGA.pdf (accessed 20.01.2017).

10. WHO. Human papillomavirus vaccines: WHO position paper, May 2017. *Releve epidemiologique hebdomadaire* 2017;92(19):241-68. [published Online First: 2017/05/23]

11. Nanteza B, Galukande M, Aceng J, et al. The burden of tetanus in Uganda. *SpringerPlus* 2016;5(1):705. doi: 10.1186/s40064-016-2309-z [published Online First: 2016/06/29]

12. Corstjens PL, Nyakundi RK, de Dood CJ, et al. Improved sensitivity of the urine CAA lateral-flow assay for diagnosing active Schistosoma infections by using larger sample volumes. *Parasites & vectors* 2015;8:241. doi: 10.1186/s13071-015-0857-7 [published Online First: 2015/04/22]

13. Webb EL, Nampijja M, Kaweesa J, et al. Helminths are positively associated with atopy and wheeze in Ugandan fishing communities: results from a cross-sectional survey. *Allergy* 2016 doi: 10.1111/all.12867

14. WHO. Haemoglobin concentrations for the diagnosis of anaemia and assessment of severity. Vitamin and Mineral Nutrition Information System. Geneva, World Health Organization, 2011 (WHO/NMH/NHD/MNM/11.1).

15. CDC. Centers for Disease Control and Prevention, vaccines and immunizations.

16. WHO. Information sheet observed rate of vaccine reactions Bacille Calmette-Guérin (BCG) vaccine. 2012

17. Arts RJW, Moorlag S, Novakovic B, et al. BCG Vaccination Protects against Experimental Viral Infection in Humans through the Induction of Cytokines Associated with Trained Immunity. *Cell host & microbe* 2018;23(1):89-100.e5. doi: 10.1016/j.chom.2017.12.010 [published Online First: 2018/01/13]
