# Supplementary material for: Impact of BCG revaccination on the response to unrelated vaccines in a Ugandan adolescent birth cohort: randomised controlled trial protocol C for the ‘POPulation differences in VACcine responses’ (POPVAC) programme
Source: BMJ Open. 2021 Feb 16;11(2):e040430. doi: 10.1136/bmjopen-2020-040430 (PMC7893605; doi:10.1136/bmjopen-2020-040430)
Supplement: Author's manuscript [file bmjopen-2020-040430.draft_revisions.pdf]

BMJ Open is committed to open peer review. As part of this commitment we make the peer review history of every article we publish publicly available.

When an article is published we post the peer reviewers' comments and the authors' responses online. We also post the versions of the paper that were used during peer review. These are the versions that the peer review comments apply to.

The versions of the paper that follow are the versions that were submitted during the peer review process. They are not the versions of record or the final published versions. They should not be cited or distributed as the published version of this manuscript.

BMJ Open is an open access journal and the full, final, typeset and author-corrected version of record of the manuscript is available on our site with no access controls, subscription charges or pay-per-view fees (http://bmjopen.bmj.com).

If you have any questions on BMJ Open's open peer review process please email info.bmjopen@bmj.com

BMJ Open

The impact of Bacillus Calmette-Guérin “pre-immunisation” on the response to unrelated vaccines in a Ugandan adolescent birth cohort: randomised controlled trial protocol C for the ‘POPulation differences in VACcine responses’ (POPVAC) programme

Journal:	*BMJ Open*
Manuscript ID	bmjopen-2020-040430
Article Type:	Protocol
Date Submitted by the Author:	14-May-2020

Complete List of Authors:	Zirimenya , Ludoviko ; Medical Research Council/Uganda Virus Research Institute and London School of Hygiene and Tropical Medicine (MRC/UVRI and LSHTM) Uganda Research Unit, Immunomodulation and Vaccines Programme<br>Nkurunungi, Gyaviira; Medical Research Council/Uganda Virus Research Institute and London School of Hygiene and Tropical Medicine (MRC/UVRI and LSHTM) Uganda Research Unit, Immunomodulation and Vaccines Programme<br>Nassuuna , Jacent; Medical Research Council/Uganda Virus Research Institute and London School of Hygiene and Tropical Medicine (MRC/UVRI and LSHTM) Uganda Research Unit, Immunomodulation and Vaccines Programme<br>Natukunda , Agnes ; Medical Research Council/Uganda Virus Research Institute and London School of Hygiene and Tropical Medicine (MRC/UVRI and LSHTM) Uganda Research Unit, Immunomodulation and Vaccines Programme<br>Mutebe , Alex ; Medical Research Council/Uganda Virus Research Institute and London School of Hygiene and Tropical Medicine (MRC/UVRI and LSHTM) Uganda Research Unit, Immunomodulation and Vaccines Programme<br>Oduru, Gloria; Medical Research Council/Uganda Virus Research Institute and London School of Hygiene and Tropical Medicine (MRC/UVRI and LSHTM) Uganda Research Unit, Immunomodulation and Vaccines Programme<br>Kabami , Grace ; Medical Research Council/Uganda Virus Research Institute and London School of Hygiene and Tropical Medicine (MRC/UVRI and LSHTM) Uganda Research Unit, Immunomodulation and Vaccines Programme<br>Akurut, Hellen; Medical Research Council/Uganda Virus Research Institute and London School of Hygiene and Tropical Medicine (MRC/UVRI and LSHTM) Uganda Research Unit, Immunomodulation and Vaccines Programme<br>Onen, Caroline; Medical Research Council/Uganda Virus Research Institute and London School of Hygiene and Tropical Medicine (MRC/UVRI and LSHTM) Uganda Research Unit, Immunomodulation and Vaccines Programme<br>Namutebi , Milly ; Medical Research Council/Uganda Virus Research

Institute and London School of Hygiene and Tropical Medicine (MRC/UVRI and LSHTM) Uganda Research Unit, Immunomodulation and Vaccines Programme
Serubanja, Joel; Medical Research Council/Uganda Virus Research Institute and London School of Hygiene and Tropical Medicine (MRC/UVRI and LSHTM) Uganda Research Unit, Immunomodulation and Vaccines Programme
Nakazibwe, Esther; Medical Research Council/Uganda Virus Research Institute and London School of Hygiene and Tropical Medicine (MRC/UVRI and LSHTM) Uganda Research Unit, Immunomodulation and Vaccines Programme
Akello , Florence ; Medical Research Council/Uganda Virus Research Institute and London School of Hygiene and Tropical Medicine (MRC/UVRI and LSHTM) Uganda Research Unit, Immunomodulation and Vaccines Programme
Tumusiime, Josephine; Medical Research Council/Uganda Virus Research Institute and London School of Hygiene and Tropical Medicine (MRC/UVRI and LSHTM) Uganda Research Unit, Immunomodulation and Vaccines Programme
Sewankambo, Moses; Medical Research Council/Uganda Virus Research Institute and London School of Hygiene and Tropical Medicine (MRC/UVRI and LSHTM) Uganda Research Unit, Immunomodulation and Vaccines Programme
Kiwanuka, Samuel; Medical Research Council/Uganda Virus Research Institute and London School of Hygiene and Tropical Medicine (MRC/UVRI and LSHTM) Uganda Research Unit, Immunomodulation and Vaccines Programme
Kiwudhu, Fred; Medical Research Council/Uganda Virus Research Institute and London School of Hygiene and Tropical Medicine (MRC/UVRI and LSHTM) Uganda Research Unit, Immunomodulation and Vaccines Programme
Kizindo , Robert ; Medical Research Council/Uganda Virus Research Institute and London School of Hygiene and Tropical Medicine (MRC/UVRI and LSHTM) Uganda Research Unit, Immunomodulation and Vaccines Programme
Kizza, Moses; Medical Research Council/Uganda Virus Research Institute and London School of Hygiene and Tropical Medicine (MRC/UVRI and LSHTM) Uganda Research Unit, Immunomodulation and Vaccines Programme
Wajja, Anne; Medical Research Council/Uganda Virus Research Institute and London School of Hygiene and Tropical Medicine (MRC/UVRI and LSHTM) Uganda Research Unit, Immunomodulation and Vaccines Programme
Cose , Stephen ; Medical Research Council/Uganda Virus Research Institute and London School of Hygiene and Tropical Medicine (MRC/UVRI and LSHTM) Uganda Research Unit, Immunomodulation and Vaccines Programme; London School of Hygiene and Tropical Medicine Department of Clinical Research
Muwanga, Moses; Entebbe Hospital
Webb, Emily; London School of Hygiene and Tropical Medicine, MRC Tropical Epidemiology Group, Department of Infectious Disease Epidemiology
Elliott, Alison ; Medical Research Council/Uganda Virus Research Institute and London School of Hygiene and Tropical Medicine (MRC/UVRI and LSHTM) Uganda Research Unit, Immunomodulation and Vaccines Programme; London School of Hygiene and Tropical Medicine Department of Clinical Research

Keywords: Infection control < INFECTIOUS DISEASES, Public health < INFECTIOUS DISEASES, Immunology < TROPICAL MEDICINE, Epidemiology < TROPICAL MEDICINE, Paediatric infectious disease & immunisation <

	PAEDIATRICS

SCHOLARONE™
Manuscripts

I, the Submitting Author has the right to grant and does grant on behalf of all authors of the Work (as defined in the below author licence), an exclusive licence and/or a non-exclusive licence for contributions from authors who are: i) UK Crown employees; ii) where BMJ has agreed a CC-BY licence shall apply, and/or iii) in accordance with the terms applicable for US Federal Government officers or employees acting as part of their official duties; on a worldwide, perpetual, irrevocable, royalty-free basis to BMJ Publishing Group Ltd ("BMJ") its licensees and where the relevant Journal is co-owned by BMJ to the co-owners of the Journal, to publish the Work in this journal and any other BMJ products and to exploit all rights, as set out in our licence.

The Submitting Author accepts and understands that any supply made under these terms is made by BMJ to the Submitting Author unless you are acting as an employee on behalf of your employer or a postgraduate student of an affiliated institution which is paying any applicable article publishing charge ("APC") for Open Access articles. Where the Submitting Author wishes to make the Work available on an Open Access basis (and intends to pay the relevant APC), the terms of reuse of such Open Access shall be governed by a Creative Commons licence – details of these licences and which Creative Commons licence will apply to this Work are set out in our licence referred to above.

Other than as permitted in any relevant BMJ Author's Self Archiving Policies, I confirm this Work has not been accepted for publication elsewhere, is not being considered for publication elsewhere and does not duplicate material already published. I confirm all authors consent to publication of this Work and authorise the granting of this licence.

For peer review only

The impact of Bacillus Calmette-Guérin "pre-immunisation" on the response to unrelated vaccines in a Ugandan adolescent birth cohort: randomised controlled trial protocol C for the 'POPulation differences in VACcine responses' (POPVAC) programme

Ludoviko Zirimenya[1,¶], Gyaviira Nkurunungi[1,¶,*], Jacent Nassuuna[1,¶], Agnes Natukunda[1,¶], Alex Mutebe[1], Gloria Oduru[1], Grace Kabami[1], Hellen Akurut[1], Caroline Onen[1], Milly Namutebi[1], Joel Serubanja[1], Esther Nakazibwe[1], Florence Akello[1], Josephine Tumusiime[1], Moses Sewankambo[1], Samuel Kiwanuka[1], Fred Kiwudhu[1], Robert Kizindo[1], Moses Kizza[1], Anne Wajja[1], Stephen Cose[1,2], Moses Muwanga[3], Emily L Webb[4], Alison M Elliott[1,2] **for the POPVAC trial team**

[1]Immunomodulation and Vaccines Programme, Medical Research Council/Uganda Virus Research Institute and London School of Hygiene and Tropical Medicine (MRC/UVRI and LSHTM) Uganda Research Unit, Entebbe, Uganda

[2]Department of Clinical Research, London School of Hygiene and Tropical Medicine, London, United Kingdom

[3]Entebbe Hospital, Entebbe, Uganda

[4]MRC Tropical Epidemiology Group, Department of Infectious Disease Epidemiology, London School of Hygiene and Tropical Medicine, London, United Kingdom

¶These authors contributed equally

***Correspondence:** Gyaviira Nkurunungi; Gyaviira.Nkurunungi@mrcuganda.org

For peer review only

Abstract

Introduction

There is evidence that Bacillus Calmette–Guérin (BCG) immunisation may protect against unrelated infectious illnesses. This has led to the postulation that administering BCG before unrelated vaccines may enhance responses to these vaccines. This might also model effects of BCG on unrelated infections.

Methods and analysis

To test this hypothesis, we have designed a randomised controlled trial of BCG versus no BCG immunisation to determine the effect of BCG on subsequent unrelated vaccines, among 300 adolescents (ages 13 to 17 years) from a Ugandan birth cohort. Our schedule will comprise three main immunisation days (week 0, week 4 and week 28): BCG (or no BCG) pre-immunisation at week 0, Yellow fever (YF-17D), Oral typhoid (Ty21a) and HPV prime at week 4, HPV boost and Tetanus/diphtheria (Td) boost at week 28. Primary outcomes are anti-YF-17D neutralising antibody titres, *Salmonella typhi* lipopolysaccharide (LPS)-specific IgG concentration, IgG specific for L1-proteins of HPV-16/18 and tetanus and diphtheria toxoid-specific IgG concentration, all assessed at four weeks after immunisation with YF, Ty21a, HPV and Td, respectively. Secondary analyses will determine effects on correlates of protective immunity (where recognised correlates exist), on vaccine response waning and on whether there are differential effects on priming vs boosting immunisations. We will also conduct exploratory immunology assays among subsets of participants to further characterise effects of BCG pre-immunisation on vaccine responses. Further analyses will assess which life-course exposures influence vaccine responses in adolescence.

Ethics and dissemination

Ethics approval has been obtained from relevant Ugandan and UK ethics committees. Results will be shared with Uganda Ministry of Health, relevant district councils, community leaders and study participants. Further dissemination will be done through conference proceedings and publications.

Trial registration

Current Controlled Trials identifier: ISRCTN10482904

Article summary

Strengths and limitations of this study

For peer review only

For peer review only - http://bmjopen.bmj.com/site/about/guidelines.xhtml

- This will be the first well-powered trial to investigate effects of BCG pre-immunisation on responses to unrelated vaccines in adolescents.
- Effects on both live-attenuated and inert vaccines will be studied.
- Our robust immunoepidemiological design and nested immunological studies will address specific hypotheses regarding pathways of effects of BCG pre-immunisation on unrelated vaccine responses.
- One limitation is that interaction between the three vaccines administered together one month after BCG immunisation may mask the true effect of BCG pre-immunisation on individual vaccine responses.

Word count

2780

Keywords

Vaccine; BCG; Immunization; Uganda

For peer review only

Introduction

There is increasing evidence that Bacillus Calmette–Guérin (BCG) immunisation has non-specific, protective effects relating to infections other than tuberculosis.[1-3] Experimental studies using BCG suggest that effects on the innate immune response are an important component of this phenomenon: BCG immunisation induces lasting epigenetic modification of innate immune cells, including monocytes, macrophages and natural killer cells.[4 5] This process, by which the innate immune system develops a form of memory, has been called "trained innate immunity".[6] Evidence is accumulating that a range of stimuli including bacterial products (particularly *Salmonella typhi* lipopolysaccharide (LPS)), and infections including malaria and hepatitis B,[7] may induce trained innate immunity; that the profile into which cells are trained varies with the dose and characteristics of the stimulus; and that effects may be induced prenatally (on exposure to maternal infections) as well as later in life.[6]

It is plausible that variation in the intensity and spectrum of experience of previous infections, and hence the epigenetic programming and consequent functional profiles of innate immune cells, contributes to the many differences in immunological activity observed between geographically and environmentally distinct settings, and hence to differences in vaccine response. If this hypothesis is correct, BCG immunisation can act as a model for the effects of prior infection, and may also be a tool for inducing enhanced benefits for other vaccines. Vaccine-specific responses can also act as a model for responses to infection. This is especially relevant given the current interest in the potential benefit of BCG immunisation against COVID-19 disease.[8 9]

In Europe, BCG "pre-immunisation" two weeks before giving influenza vaccine has been shown to result in enhanced antibody responses to influenza proteins.[10] BCG "pre-immunisation" four weeks before giving Yellow Fever (YF 17D) vaccine has also been found to result in reduced replication of the yellow fever vaccine virus; this was not associated with a significant reduction in the desired neutralising antibody response to YF, or in the interferon (IFN)-γ response, but the study size was small and may not have had sufficient power to demonstrate important effects.[11]

In Uganda, BCG immunisation at birth is recommended.[12] The benefits of BCG immunisation in adolescence for protection against tuberculosis are not known and may differ between settings.[13] Whether BCG immunisation in adolescents in Uganda will have non-specific effects on the innate immune response, on subsequent immunisations and (indeed) on general health (given the prior exposure at birth, and the on-going exposure to non-tuberculous mycobacteria and other infections) is not known. In Protocol C of the 'Population differences in Vaccine responses" programme

For peer review only - http://bmjopen.bmj.com/site/about/guidelines.xhtml

96 (POPVAC C; Current Controlled Trials identifier: ISRCTN10482904), we plan to address this knowledge gap by randomising adolescent members of the Entebbe Mother and Baby Study (EMaBS) birth cohort[12] in a nested trial of BCG "pre-immunisation" versus no BCG immunisation prior to immunisation with other vaccines. We summarise the protocol here.

For peer review only

100 **Hypothesis**

101 The overarching goal of the POPVAC programme is to understand population differences in vaccine
102 responses in Uganda, in order to identify strategies through which vaccine effectiveness can be
103 optimised for the low-income, tropical settings where they are especially needed. For this Trial C we
104 address the concept of trained innate immunity through the hypothesis that BCG "pre-
105 immunisation" modifies the response to subsequent unrelated vaccines.

106 **Objective**

107 To determine whether BCG "pre-immunisation" modulates the response to unrelated vaccines
108 among Ugandan adolescents.

For peer review only

Methods and analysis

Setting and participants

SPIRIT reporting guidelines[14] are used. This trial will be a randomised, controlled, open, parallel group trial investigating the effect of BCG "pre-immunisation" on unrelated vaccine response outcomes. The study will take place in Entebbe municipality, Wakiso District, Uganda and will involve participants in the EMaBS birth cohort.[12] In EMaBS, a cohort of 2500 pregnant women were recruited between 2003 and 2005 for a trial of anthelminthic treatment during pregnancy and early childhood, investigating effects on childhood vaccine responses and infectious disease incidence.[12] We aim to enroll 300 of the EMaBS birth cohort participants, randomising 150 to each intervention arm. All EMaBS participants received BCG at birth; hence current trial participants (in the BCG intervention arm) will undergo revaccination. EMaBS participants are expected to be aged 13 to 17 during recruitment to this study.

Recruitment criteria

Inclusion criteria

i. A participant in the Entebbe Mother and Baby Study[12]
ii. Written informed consent by parent or guardian
iii. Written informed assent by participant
iv. Willing to remain in the study area for the duration of the study
v. Willing to provide locator information and to be contacted during the course of the trial
vi. Females agree to avoid pregnancy for the duration of the trial
vii. Able and willing (in the investigator's opinion) to comply with all the study requirements

Exclusion criteria

i. Concurrent enrolment into another clinical trial
ii. Clinically significant history of immunodeficiency (including HIV), cancer, cardiovascular disease, gastrointestinal disease, liver disease, renal disease, endocrine disorder and neurological illness
iii. History of serious psychiatric condition or disorder
iv. Moderate or severe acute illness characterised by any of the following symptoms: fever, impaired consciousness, convulsions, difficulty in breathing, vomiting; or as determined by the attending project clinician.

For peer review only

For peer review only - http://bmjopen.bmj.com/site/about/guidelines.xhtml

v. History of previous immunisation with Yellow Fever (YF), oral typhoid or Human Papillomavirus (HPV) vaccine; previous immunisation with BCG or Tetanus and diphtheria vaccine (Td) at age $\geq$5 years

vi. Concurrent oral or systemic steroid medication or the concurrent use of other immunosuppressive agents within 2 months prior to enrolment

vii. History of allergic reaction to immunisation or any allergy likely to be exacerbated by any component of the study vaccines including egg or chicken proteins

viii. Tendency to develop keloid scars

ix. Positive HIV serology

x. Positive pregnancy test

xi. Female currently lactating, confirmed pregnancy or intention to become pregnant during the trial period

xii. Use of an investigational medicinal product or non-registered drug, live vaccine, or medical device other than the study vaccines for 30 days prior to dosing with the study vaccine, or planned use during the study period

xiii. Administration of immunoglobulins and/or any blood products within the three months preceding the planned trial immunisation date

Interventions

We will randomise participants to receive BCG or not to receive BCG, four weeks prior to immunisation with a panel of licensed unrelated vaccines (discussed below). The adolescents in the intervention arm will receive a dose of 0.1 ml in the deltoid region of the right upper arm.

Randomisation and allocation to treatment arm

An independent statistician will generate the randomisation code using a randomly permuted block size. This code will be embedded as a web-based randomisation system in REDCap (Research Electronic Data Capture) software.[15 16] Randomisation to the two trial arms will be done in a 1:1 ratio. At enrolment, eligibility criteria will be checked and eligible participants will be allocated sequentially to the next randomisation number, with the corresponding trial arm designated in REDCap. The randomisation code will be kept securely by the trial statistician with a second copy held by a data manager or statistician not otherwise involved in the trial at the MRC/UVRI and LSHTM Uganda Research Unit.

Blinding

This trial will not be blinded to clinicians or participants since they will not participate in outcome ascertainment and the expected development of a BCG scar makes blinding difficult. It is unlikely

For peer review only

For peer review only - http://bmjopen.bmj.com/site/about/guidelines.xhtml

that participants allocated to “no BCG” will seek this privately. Only laboratory personnel evaluating vaccine response outcomes will be unaware of BCG allocation so outcome ascertainment will not be biased through lack of blinding.

Immunisations

We anticipate that BCG pre-immunisation may have different effects on live and non-live, oral and parenteral, priming and boosting vaccines. Activated innate responses may kill live vaccines and suppress subsequent adaptive responses by this, or other, mechanisms,[17 18] but bias, or even enhance, responses to toxoids or proteins;[19-21] thus, results from a single-vaccine study would not be generalisable.

We therefore propose to study a portfolio of licensed vaccines (live and inert, oral and parental, priming and boosting) expected to be beneficial (in some cases, already given) to adolescents in Uganda. Our schedule (**Table 1** and supplementary **Table S1**) will comprise three main immunisation days (week 0, week 4 and week 28). Additional HPV immunisation will be provided for girls aged 14 years or above, and a second Td boost will be given after completion of the study, to accord with the national Expanded Programme on Immunisation (EPI) routines but the response to these will not specifically be addressed. Further rationale for the selection of vaccines is detailed in supplementary information. Our schedule has been developed in consultation with the EPI programme and is cognizant of potential interference between vaccines.

Table 1. Immunisation schedule

	**Immunisation week 0**	**Immunisation week 4**	**[Immunisation week 8]**	**Immunisation week 28**	**[Immunisation week 52]**
**Live vaccines**	BCG re-vaccination[1]	Yellow fever (YF-17D)<br>Oral typhoid (Ty21a)			
**Non-live vaccines**		HPV prime	HPV boost for girls aged ≥14 years[2,3]	HPV boost and Tetanus/ diphtheria (Td) boost	Tetanus/ diphtheria (Td) boost[3,4]

1. Prior BCG status may vary (data on history and documentation of prior BCG, and presence of a BCG scar, will be documented although these approaches have limitations for determining BCG status)
2. The National EPI programme recommends three doses of HPV vaccine for older girls
3. These doses will be given to comply with guidelines but outcomes specifically relating to these doses will not be assessed
4. Priming by immunisation in infancy is assumed

For peer review only

Schedule of immunisation and sampling

The schedule of immunisation and sampling is outlined in **Table S1**. While optimal timings for outcome measures vary between vaccines, sampling at 8 weeks post BCG and 4 weeks post YF-17D, Ty21a, HPV and Td is proposed for the primary endpoints, targeting the establishment of memory responses and approximate peak of antibody responses. A secondary endpoint at one year will assess waning. All analyses will take baseline measurements into account. Immunisation postponement criteria are detailed in Supplementary information.

Outcomes

Primary outcomes

These will be assessed in all participants.

i. **YF-17D**: neutralising antibody titres (plaque-reduction neutralisation test) at four weeks post YF immunisation.
ii. **Ty21a**: *Salmonella typhi* lipopolysaccharide (LPS)-specific immunoglobulin (Ig)G concentration at four weeks post Ty21a immunisation.
iii. **HPV:** IgG specific for L1-proteins of HPV-16/18 at four weeks post HPV priming immunisation.
iv. **Td:** tetanus and diphtheria toxoid-specific IgG concentration at four weeks post Td immunisation.

Secondary outcomes

These will be assessed in all participants and will further investigate estimates of protective immunity (for vaccines where these are available) and dynamics of the vaccine responses, as well as the impact of the interventions on parasite clearance.

i. **Protective immunity**: Proportions with protective neutralising antibody (YF); protective IgG levels (TT);[22] seroconversion rates (Ty21a) at four weeks post the corresponding immunisation.
ii. **Response waning**: Primary outcome measures (all vaccines) repeated at week 52, and area-under-the curve (AUC) analyses. Parasitic infection may accelerate,[23] and anti-parasitic interventions delay, waning.
iii. **Priming versus boosting**: Effects on priming versus boosting will be examined for HPV only, comparing outcomes four weeks after the first, and four weeks after the second vaccine dose.

For peer review only - http://bmjopen.bmj.com/site/about/guidelines.xhtml

Furthermore, our sample collection will offer opportunities for an array of exploratory immunological evaluations on stored samples, focusing mainly on vaccine antigen specific outcomes. Exploratory assays will provide further detail on the mechanisms underlying effects of BCG on responses to unrelated vaccines.

Additional measurements

Other additional assays are discussed in Supplementary information, and will comprise evaluation of helminth and malaria infection exposure, HIV serology (at baseline), pregnancy and full blood count testing (at baseline and before immunisation on each immunisation day).

Sample size considerations

Based on the literature[17 24 25] and preliminary data, we anticipate that standard deviations (SDs) of primary outcome measures will lie between 0.3 and 0.6 $\log_{10}$; and that pre-immunisation with BCG may increase responses by approximately 0.12-0.14 $\log_{10}$. Based on these assumptions, we aim to enrol 300 EMaBS participants (150 BCG “pre-immunisation”, 150 no BCG immunisation). Allowing for 10% loss to follow-up, this will give over 90% power to detect a difference of 0.12$\log_{10}$ in vaccine response between the pre-BCG immunised and non-pre-immunised groups, at 5% significance level and assuming vaccine response standard deviation of 0.3$\log_{10}$.

Table 2. Power estimates (5% significance level)

	**Log_{10} difference**						
**Standard deviation ($\log_{10}$)**	**0.08**	**0.10**	**0.12**	**0.14**	**0.16**	**0.18**	**0.20**
Trial C: 150 BCG “pre-immunisation” vs 150 no BCG immunisation							
0.3	59%	78%	91%	97%	99%	>99%	>99%
0.4	37%	53%	69%	82%	91%	96%	98%
0.5	26%	37%	50%	63%	75%	84%	91%
0.6	19%	28%	37%	48%	59%	69%	78%

Cells highlighted in grey correspond to >80% power.

Ethical and regulatory considerations

Ethical approval has been granted from the Research Ethics Committees of the Uganda Virus Research Institute (reference: GC/127/19/05/682), the London School of Hygiene and Tropical Medicine (reference: 16034), the Uganda National Council for Science and Technology (reference: HS 2491) and from the Uganda National Drug Authority (certificate number: CTA0094). Any protocol amendments will be submitted to ethics committees and regulatory bodies for approval before implementation.

For peer review only - http://bmjopen.bmj.com/site/about/guidelines.xhtml

Participants will be adolescents and therefore a vulnerable human population. Care will be taken to provide adequate, age- and education-status appropriate information and to ensure that it is understood; and to emphasise that participation is voluntary. Participants will be enrolled only when they have given their own assent and when consent has been given by the parent or guardian. No major risks to the participants are anticipated since all the vaccines to be given are licensed and known to be safe.

With regard to BCG immunisation or revaccination in adolescence, benefits with respect to protection against tuberculosis among Ugandan adolescents are unknown and may, at best, be modest. There may be non-specific benefits. WHO's SAGE committee concluded, in their summary of October 2017,[26] that "BCG revaccination is safe in *Mycobacterium tuberculosis* infected and uninfected populations. There is a lack of evidence from randomised controlled trials and retrospective cohort and case-control studies demonstrating the efficacy and effectiveness of BCG revaccination in adolescents and adults after primary BCG vaccination in infancy for protection against TB disease. Due to absence of evidence, BCG revaccination is not considered cost-effective. Further research is warranted to explore whether certain sub-groups of age, geographic or *M. tuberculosis* exposure categories would benefit from BCG revaccination." We hope, through this work, to contribute to this debate.

Patient and public involvement

The EMaBS research team has previously worked with volunteer local council field workers to ensure regular follow up of participants and these field workers continue to attend participants' meetings and provide a mechanism by which the communities from which participants are drawn can be informed about on-going work. As well, prior to the start of this study, we will share our plans with district health and education officers, and with colleagues at Entebbe Hospital. We will establish an advisory committee of parents who will help us to ensure that EMaBS cohort members can participate in the study without undue disruption to their school work. Study findings will be shared with these stakeholders and with participants.

Dissemination

Study findings will be published through open access peer-reviewed journals, presentations at local, national and international conferences and to the local community through community meetings. Anonymised participant level datasets generated will be available upon request.

Data management and analysis

Socio-demographic information and clinical and laboratory measurements will be recorded and managed using REDCap (Research Electronic Data Capture) tools,[15 16] with paper-based forms as

For peer review only - http://bmjopen.bmj.com/site/about/guidelines.xhtml

280 back-up. All data will be recorded under a unique study ID number. When paper forms must be
281 used, data will be double entered in a study-specific database, with standard checks for
282 discrepancies. All data for analysis will be anonymised and stored on a secure and password-
283 protected server, with access limited to essential research personnel.

284 The effect of BCG versus no BCG pre-immunisation on the outcomes will be analysed. The analysis
285 will test whether BCG pre-immunisation alters the response to live or inert vaccines given four
286 weeks later, including effects on vaccine replication, immune response profile, priming, boosting and
287 waning. It will indicate whether including BCG as a component of school-based immunisation
288 schedules is likely to have non-specific benefits for Ugandan adolescents.

For peer review only

For peer review only - http://bmjopen.bmj.com/site/about/guidelines.xhtml

Discussion

It is increasingly clear that several live vaccines, including BCG, measles vaccine and Vaccinia (smallpox) vaccine, have non-specific, generally beneficial, effects including reduced mortality (not related to the infectious disease that they were designed to target).[1 2] The potential effects of BCG on responses to unrelated vaccines, specifically on live-attenuated ones such as yellow fever and oral typhoid, might model its effects on responses to unrelated infectious agents. We hypothesise that BCG immunisation both achieves non-specific benefits, and influences vaccine responses, through mechanisms based on effects on the innate immune system and consequent immunological profile.

Of note, in this Ugandan birth cohort, all participants were documented to have received BCG at birth, with the strain of BCG used recorded.[12] This will therefore be the first well-powered study to investigate effects of BCG re-vaccination on vaccine responses in adolescents. This study will determine whether BCG pre-immunisation alters the response to live or inert vaccines given four weeks later, including effects on vaccine replication, immune response profile, priming, boosting and waning among adolescents who received BCG as infants. It will indicate whether including BCG as a component of school-based immunisation schedules is likely to have non-specific benefits for Ugandan adolescents and other settings where infant BCG immunisation is common. If this is correct, BCG immunisation may be used as a tool for inducing enhanced benefits for other vaccines in a wide range of settings.

Study timeline

Applications for ethical approval were submitted in May 2018, with approval received in September 2018 (Uganda Virus Research Institute Research Ethics Committee), May 2019 (National Drug Authority and Uganda National Council for Science and Technology) and June 2019 (London School of Hygiene and Tropical Medicine). Collaborator/investigator/trial steering committee meetings were also held during the initial 12-month planning period. Recruitment is scheduled to commence in May 2020. Intervention will be up to 12 months, with completion of the project scheduled for April 2022.

For peer review only - http://bmjopen.bmj.com/site/about/guidelines.xhtml

Competing interests

Alison Elliott reports a grant from the Medical research Council, UK (POPVAC programme funding). The rest of the authors declare that they have no conflicts of interest.

Author contributions

AME conceived the study. AME, GN, ELW, AN, AW, SC, LZ and MM contributed to study design. LZ, GO, GK, JS, CO, MN, EN, FA and JT are site clinicians/nurses/clinical laboratory technicians providing valuable input on clinical considerations of the intervention. MS, SK, FK, RK and MK are field workers and administrators handling the organisational integration of the intervention. AN, AM, HA and ELW are involved in organisation of the databases, trial randomization, treatment allocation and drawing up of analytical plans. LZ, GN, JN, AN, SC, ELW and AME drafted the manuscript. All authors reviewed the manuscript, contributed to it and approved the final version.

Acknowledgements

We thank the Uganda National Expanded Programme for Immunisation, Sanofi Pasteur and PaxVax for providing the HPV, yellow fever and oral typhoid vaccines, respectively. The BCG and tetanus-diphtheria vaccines were kind donations from the Serum Institute of India. We thank the Wakiso district local government and Entebbe hospital for their support. We also thank members of the POPVAC programme steering committee (chaired by Prof. Richard Hayes) and the Data and Safety Monitoring Board (Dr David Meya, Prof. Andrew Prendergast and Dr Elizabeth George).

Funding

The POPVAC programme of work is supported by the Medical Research Council of the United Kingdom (MR/R02118X/1). SC and JN were supported in part by the Makerere University – Uganda Virus Research Institute Centre of Excellence for Infection and Immunity Research and Training (MUII-plus). MUII-plus is funded under the DELTAS Africa Initiative. The DELTAS Africa Initiative is an independent funding scheme of the African Academy of Sciences (AAS), Alliance for Accelerating Excellence in Science in Africa (AESA) and supported by the New Partnership for Africa's Development Planning and Coordinating Agency (NEPAD Agency) with funding from the Wellcome Trust (grant 107743) and the UK Government. The MRC/UVRI and LSHTM Uganda Research Unit is jointly funded by the UK Medical Research Council (MRC) and the UK Department for International Development (DFID) under the MRC/DFID Concordat agreement and is also part of the EDCTP2 programme supported by the European Union.

For peer review only

For peer review only - http://bmjopen.bmj.com/site/about/guidelines.xhtml

The study sponsor (London School of Hygiene and Tropical Medicine) and funders had no role in study design; collection, management, analysis, and interpretation of data; writing of the protocol; and the decision to submit the protocol for publication.

POPVAC trial team

Principal investigator*:* Alison Elliott; ***Project leader***: Ludoviko Zirimenya; ***laboratory staff****:* Gyaviira Nkurunungi, Stephen Cose, Rebecca Amongin, Beatrice Nassanga, Jacent Nassuuna, Irene Nambuya, Prossy Kabuubi, Emmanuel Niwagaba, Gloria Oduru, Grace Kabami; ***statisticians and data managers****:* Emily Webb, Agnes Natukunda, Helen Akurut, Alex Mutebe; ***clinicians****:* Anne Wajja, Milly Namutebi, Christopher Zziwa, Joel Serubanja; ***nurses****:* Caroline Onen, Esther Nakazibwe, Josephine Tumusiime, Caroline Ninsiima, Susan Amongi, Florence Akello; ***internal monitor****:* Mirriam Akello; ***field workers****:* Robert Kizindo, Moses Sewankambo, Denis Nsubuga, Samuel Kiwanuka, Fred Kiwudhu; ***boatman****:* David Abiriga; ***administrative management****:* Moses Kizza, Samsi Nansukusa; ***internal and external collaborators:*** Pontiano Kaleebu, Hermelijn Smits, Maria Yazdanbakhsh, Govert van Dam, Paul Corstjens, Sarah Staedke, Henry Luzze, James Kaweesa, Edridah Tukahebwa, Elly Tumushabe, Moses Muwanga.

For peer review only

For peer review only - http://bmjopen.bmj.com/site/about/guidelines.xhtml

REFERENCES

1. Benn CS, Netea MG, Selin LK, et al. A small jab - a big effect: nonspecific immunomodulation by vaccines. *Trends in immunology* 2013;34(9):431-9. doi: 10.1016/j.it.2013.04.004 [published Online First: 2013/05/18]

2. Rieckmann A, Villumsen M, Sorup S, et al. Vaccinations against smallpox and tuberculosis are associated with better long-term survival: a Danish case-cohort study 1971-2010. *International journal of epidemiology* 2017;46(2):695-705. doi: 10.1093/ije/dyw120 [published Online First: 2016/07/07]

3. Prentice S, Webb EL, Dockrell HM, et al. Investigating the non-specific effects of BCG vaccination on the innate immune system in Ugandan neonates: study protocol for a randomised controlled trial. *Trials* 2015;16:149. doi: 10.1186/s13063-015-0682-5

4. Blok BA, Arts RJ, van Crevel R, et al. Trained innate immunity as underlying mechanism for the long-term, nonspecific effects of vaccines. *J Leukoc Biol* 2015;98(3):347-56. doi: 10.1189/jlb.5RI0315-096R

5. Kleinnijenhuis J, Quintin J, Preijers F, et al. Bacille Calmette-Guerin induces NOD2-dependent nonspecific protection from reinfection via epigenetic reprogramming of monocytes. *Proceedings of the National Academy of Sciences of the United States of America* 2012;109(43):17537-42. doi: 10.1073/pnas.1202870109

6. Rusek P, Wala M, Druszczynska M, et al. Infectious Agents as Stimuli of Trained Innate Immunity. *International journal of molecular sciences* 2018;19(2) doi: 10.3390/ijms19020456 [published Online First: 2018/02/07]

7. Schrum JE, Crabtree JN, Dobbs KR, et al. Cutting Edge: Plasmodium falciparum Induces Trained Innate Immunity. *Journal of immunology (Baltimore, Md : 1950)* 2018;200(4):1243-48. doi: 10.4049/jimmunol.1701010 [published Online First: 2018/01/14]

8. Dayal D, Gupta S. Connecting BCG Vaccination and COVID-19: Additional Data. *medRxiv* 2020:2020.04.07.20053272. doi: 10.1101/2020.04.07.20053272

9. Miller A, Reandelar MJ, Fasciglione K, et al. Correlation between universal BCG vaccination policy and reduced morbidity and mortality for COVID-19: an epidemiological study. *medRxiv* 2020:2020.03.24.20042937. doi: 10.1101/2020.03.24.20042937

10. Leentjens J, Kox M, Stokman R, et al. BCG Vaccination Enhances the Immunogenicity of Subsequent Influenza Vaccination in Healthy Volunteers: A Randomized, Placebo-Controlled Pilot Study. *The Journal of infectious diseases* 2015;212(12):1930-8. doi: 10.1093/infdis/jiv332

11. Arts RJW, Moorlag S, Novakovic B, et al. BCG Vaccination Protects against Experimental Viral Infection in Humans through the Induction of Cytokines Associated with Trained Immunity. *Cell host & microbe* 2018;23(1):89-100.e5. doi: 10.1016/j.chom.2017.12.010 [published Online First: 2018/01/13]

12. Webb EL, Mawa PA, Ndibazza J, et al. Effect of single-dose anthelmintic treatment during pregnancy on an infant's response to immunisation and on susceptibility to infectious diseases in infancy: a randomised, double-blind, placebo-controlled trial. *Lancet* 2011;377(9759):52-62. doi: 10.1016/S0140-6736(10)61457-2 [published Online First: 2010/12/24]

13. Barreto ML, Pilger D, Pereira SM, et al. Causes of variation in BCG vaccine efficacy: examining evidence from the BCG REVAC cluster randomized trial to explore the masking and the blocking hypotheses. *Vaccine* 2014;32(30):3759-64. doi: 10.1016/j.vaccine.2014.05.042

14. Chan AW, Tetzlaff JM, Altman DG, et al. SPIRIT 2013 statement: defining standard protocol items for clinical trials. *Ann Intern Med* 2013;158(3):200-7. doi: 10.7326/0003-4819-158-3-201302050-00583 [published Online First: 2013/01/09]

For peer review only

For peer review only - http://bmjopen.bmj.com/site/about/guidelines.xhtml

15. Harris PA, Taylor R, Minor BL, et al. The REDCap Consortium: Building an International Community of Software Platform Partners. *Journal of biomedical informatics* 2019:103208. doi: 10.1016/j.jbi.2019.103208 [published Online First: 2019/05/13]

16. Harris PA, Taylor R, Thielke R, et al. Research electronic data capture (REDCap)--a metadata-driven methodology and workflow process for providing translational research informatics support. *Journal of biomedical informatics* 2009;42(2):377-81. doi: 10.1016/j.jbi.2008.08.010 [published Online First: 2008/10/22]

17. Muyanja E, Ssemaganda A, Ngauv P, et al. Immune activation alters cellular and humoral responses to yellow fever 17D vaccine. *The Journal of clinical investigation* 2014;124(7):3147-58. doi: 10.1172/jci75429 [published Online First: 2014/06/10]

18. Akondy RS, Johnson PL, Nakaya HI, et al. Initial viral load determines the magnitude of the human CD8 T cell response to yellow fever vaccination. *Proceedings of the National Academy of Sciences of the United States of America* 2015;112(10):3050-5. doi: 10.1073/pnas.1500475112 [published Online First: 2015/02/26]

19. Sabin EA, Araujo MI, Carvalho EM, et al. Impairment of tetanus toxoid-specific Th1-like immune responses in humans infected with Schistosoma mansoni. *The Journal of infectious diseases* 1996;173(1):269-72. [published Online First: 1996/01/01]

20. Elliott AM, Mawa PA, Webb EL, et al. Effects of maternal and infant co-infections, and of maternal immunisation, on the infant response to BCG and tetanus immunisation. *Vaccine* 2010;29(2):247-55. doi: 10.1016/j.vaccine.2010.10.047

21. Brown J, Baisley K, Kavishe B, et al. Impact of malaria and helminth infections on immunogenicity of the human papillomavirus-16/18 AS04-adjuvanted vaccine in Tanzania. *Vaccine* 2014;32(5):611-7. doi: 10.1016/j.vaccine.2013.11.061

22. Plotkin SA. Correlates of protection induced by vaccination. *Clinical and vaccine immunology : CVI* 2010;17(7):1055-65. doi: 10.1128/cvi.00131-10 [published Online First: 2010/05/14]

23. Riner DK, Ndombi EM, Carter JM, et al. Schistosoma mansoni Infection Can Jeopardize the Duration of Protective Levels of Antibody Responses to Immunizations against Hepatitis B and Tetanus Toxoid. *PLoS neglected tropical diseases* 2016;10(12):e0005180. doi: 10.1371/journal.pntd.0005180

24. Fletcher HA, Snowden MA, Landry B, et al. T-cell activation is an immune correlate of risk in BCG vaccinated infants. *Nat Commun* 2016;7:11290. doi: 10.1038/ncomms11290

25. Safaeian M, Porras C, Pan Y, et al. Durable antibody responses following one dose of the bivalent human papillomavirus L1 virus-like particle vaccine in the Costa Rica Vaccine Trial. *Cancer prevention research (Philadelphia, Pa)* 2013;6(11):1242-50. doi: 10.1158/1940-6207.capr-13-0203 [published Online First: 2013/11/06]

26. WHO. Immunization, vaccines and biologicals. Vaccine position papers. 2017

For peer review only - http://bmjopen.bmj.com/site/about/guidelines.xhtml

SUPPLEMENTARY INFORMATION

The impact of Bacillus Calmette-Guérin “pre-immunisation” on the response to unrelated vaccines in a Ugandan adolescent birth cohort: randomised controlled trial protocol C for the ‘POPulation differences in VACcine responses’ (POPVAC) programme

Ludoviko Zirimenya[1,¶], Gyaviira Nkurunungi[1,¶,*], Jacent Nassuuna[1,¶], Agnes Natukunda[1,¶], Alex Mutebe[1], Gloria Oduru[1], Grace Kabami[1], Hellen Akurut[1], Caroline Onen[1], Milly Namutebi[1], Joel Serubanja[1], Esther Nakazibwe[1], Florence Akello[1], Josephine Tumusiime[1], Moses Sewankambo[1], Samuel Kiwanuka[1], Fred Kiwudhu[1], Robert Kizindo[1], Anne Wajja[1], Stephen Cose[1,2], Moses Muwanga[3], Emily L Webb[4], Alison M Elliott[1,2] **for the POPVAC trial team**

[1]Immunomodulation and Vaccines Programme, Medical Research Council/Uganda Virus Research Institute and London School of Hygiene and Tropical Medicine (MRC/UVRI and LSHTM) Uganda Research Unit, Entebbe, Uganda

[2]Department of Clinical Research, London School of Hygiene and Tropical Medicine, London, United Kingdom

[3]Entebbe Hospital, Entebbe, Uganda

[4]MRC Tropical Epidemiology Group, Department of Infectious Disease Epidemiology, London School of Hygiene and Tropical Medicine, London, United Kingdom

[¶]These authors contributed equally

***Correspondence:** Gyaviira Nkurunungi; Gyaviira.Nkurunungi@mrcuganda.org

For peer review only

Table S1: Schedule of visits and procedures

VISIT NUMBER	1	2	3	3.2, 3.3	4	5	6
WEEKS FROM 1ST IMMUNISATION	-4 to 0[1]	0	4	4 weeks +4 days	8	28	52 SE
	Screening	Immunisation	Immunisations		Primary endpoint (PE)	Immunisations	Secondary endpoint (SE)
**RANDOMISED BCG "PRE-IMMUNISATION"**							
BCG arm (x)		**x**					
No BCG arm (o)		**o[2]**					
**ANTHELMINTHIC TREATMENT**							
Praziquantel and albendazole or mebendazole					**X[3]**	**X[3]**	**X[3]**
**VACCINES**							
YF-17D			x				
Ty21a			X[7]				
HPV			x		[x][4]	x	
Td						x	[x][5]
**INVESTIGATIONS/PROCEDURES**							
Inclusion/exclusion criteria	x						
Informed consent	x						
Questionnaire	x		[x][2]	x	x	x	x
Examination	x		(x)	(x)	(x)	(x)	(x)
Urine β-HCG test (female only) 1mL	x	x[6]	[x][2]			x	
Urine YF viral load				x			
Stool for PCR and storage	x						x
Stool for coproantibody and storage	x				x		
**BLOOD TESTS**							
Malaria PCR (1ml)	x						x
Serology for HIV, prior malaria and *S. mansoni* (0.5 ml)	x						
Mansonella perstans (1ml)	x						
Full blood count (1ml)	x		[x][2]				
Assessments of pre-immunisation responses, and/or vaccine response outcomes and/or exploratory immunology; storage[9] (10-20ml)	x		[x][2]		x		x
Blood for gene expression (2ml)	x		[x][2]				
Blood vol (mL)	27		17		20		25
Cumulative blood vol (mL)[8]	27		44		64		89

PE: primary endpoint; **SE:** secondary endpoint
Immunisation days highlighted in green, primary end point days in red
(**x**) performed if clinically indicated
1. Screening and enrolment into Project C will take place shortly before enrolment, sometimes on the same day

For peer review only - http://bmjopen.bmj.com/site/about/guidelines.xhtml

2. Individuals allocated to no BCG may be immunised immediately “skipping” to week 4. Procedures indicated in square brackets need not be repeated for the no BCG arm if this is done.
3. Treatments given after sample when schedules coincide
4. Week 8 HPV dose will be given for previously-unvaccinated girls aged ≥14 years
5. Week 52 Td booster dose will be provided as a service
6. Pregnancy test to be repeated if more than 4 weeks elapses between screening and immunisation
7. Oral typhoid vaccine doses will be administered on three alternate days namely visit 3, 3.1, and 3.2
8. Exploratory immunology blood volume will be guided by guidelines from Harvard Mass General, where a maximum of 3ml/kg body weight is taken at any one time point and not more than 3ml/kg is taken over any 8-week period (ref http://www.drgreene.com/21_1616.html.) These guidelines have been followed in a previous study vaccinating adolescents with investigational tuberculosis vaccine MVA85A (in Uganda).[1] The total blood volume planned is 64 ml over the initial intensive sampling period of 8 weeks. Revision of sample volumes based on weight will only be required for participants who weigh less than 21 kg; the average weight of children aged 9 years is expected to be 28kg (with 21kg the 3rd centile) with greater weights for older children.[2]

For peer review only

For peer review only - http://bmjopen.bmj.com/site/about/guidelines.xhtml

Further rationale for the selection of vaccines

Yellow fever vaccine

Yellow fever vaccine YF-17D is a live replicating parenteral vaccine. The vaccine (Stamaril; Sanofi Pasteur) is available for purchase in Uganda. Yellow Fever (YF) causes outbreaks in Uganda and the wider region[3] and YF-17D is a candidate for Uganda's expanded programme on immunisation (EPI; H Luzze, personal communication). As noted above, lower vaccine replication, lower neutralising antibody induction, and greater waning, are described in Uganda compared to Switzerland.[4] YF-17D is a potential vector for novel vaccine constructs,[5] adding relevance to vaccine development.

Typhoid vaccine Ty21a

Typhoid vaccine Ty21a is a live replicating oral vaccine and also a potential vector for new vaccine constructs.[6] Ty21a vaccine will be purchased from PaxVax, Redwood City, California. Substantial, multi-year typhoid outbreaks occur in Uganda and immunisation campaigns have been advocated as cost effective.[7]

Ty21a was developed in the 1970s. Although not routinely used in Uganda, it has been (and is currently) registered in many countries. It was first registered in the United States and United Kingdom in the 1980s, and is recommended by the World Health Organisation for both endemic and epidemic settings.[8] It has comparable efficacy to the parenteral Vi polysaccharide typhoid vaccine, good durability and minimal adverse effects.[8] It is proposed for use in this study to model effects of study exposures and intervention on the response to a live oral vaccine.

The Ty21a vaccine is given as a three-dose regimen on alternate days.

Human Papilloma Virus (HPV) vaccine

Human Papilloma Virus (HPV) vaccine is a protein virus-like particle. The quadrivalent HPV Vaccine Gardasil (Merck) is available for purchase in Uganda and is the vaccine used by the national EPI programme. HPV immunisation is being rolled out among girls to prevent cervical neoplasia, the commonest cancer among Ugandan women and we will coordinate provision with the national HPV immunisation programme.[9] HPV immunisation is also beneficial for boys since HPV infection is associated with anogenital warts, anal cancer and oropharyngeal cancers in both males and females, and with penile cancer in men,[10] and we will include boys in these studies.

Tetanus and diphtheria vaccines

Tetanus and diphtheria vaccines comprise inert toxoids (Td). Booster immunisation is recommended for young women to prevent maternal and neonatal tetanus. Recent evidence emphasises the need to protect young men also.[11]

For peer review only - http://bmjopen.bmj.com/site/about/guidelines.xhtml

Immunisation Postponement Criteria

If any one of the following is identified at the time scheduled for immunisation, the participant may be immunised at a later date, or withdrawn, at the discretion of the Investigator. The participant must be followed until resolution of the event as with any adverse event:

- Acute disease at the time of immunisation. Acute disease is defined as the presence of a moderate or severe illness with or without fever. All vaccines can be administered to persons with a minor illness such as diarrhoea or mild upper respiratory infection with or without low-grade fever, i.e. temperature of ≤37.5°C (99.5°F)
- Temperature of >37.5°C (99.5°F) at the time of immunisation
- Taking antibiotics or antimalarials currently, or within the past 7 days, of the date of Ty21a administration (ascertained verbally)

Vaccine storage and transport

In order to maintain a reliable vaccine cold chain, the vaccines and diluents to be used will be stored and transported within the recommended temperature range of +2ºC to +8ºC. Care will be taken to ensure that the vaccines are not frozen. BCG, being sensitive to light, will be kept in the dark (normally within its secondary packaging) for as long as possible to protect it during storage and transportation. All vaccines will be kept in appropriate refrigeration equipment with a temperature monitoring device to ensure temperatures remain between +2ºC and +8ºC. Cold boxes/vaccines carriers with temperature monitors will be used to transport vaccines and the diluents from the MRC/UVRI and LSHTM Uganda Research Unit (Entebbe) to the clinic where vaccination will take place and while transporting vaccines to immunisation sessions. Designated staff will be given responsibility for managing the vaccine cold chain. All cold chain equipment including the temperature monitoring devices used for this project will comply with relevant technical specifications as defined by the EPI standards. Basic routine maintenance will be regularly carried out on all cold chain equipment.

Additional laboratory measurements

Additional assays will comprise measurement of parasite infection exposure, HIV serology, pregnancy testing and full blood counts. HIV testing and pregnancy testing will be accompanied by appropriate counselling by trained staff.

Current *S. mansoni* infection status and intensity will be determined by serum/plasma levels of circulating anodic antigen (CAA). The method is quantitative, highly specific for *Schistosoma* infection, and much more sensitive than the conventional Kato Katz method.[12] CAA will be assessed retrospectively on stored samples collected at baseline.

For peer review only - http://bmjopen.bmj.com/site/about/guidelines.xhtml

Prior exposure to schistosomiasis will be evaluated by ELISA for IgG to schistosome egg antigen using stored blood samples collected at baseline.

The presence of other helminth infections will be determined retrospectively using stool PCR of samples collected at baseline and at weeks 28 and 52.[13] In accordance with national guidelines, all participants will be treated with albendazole or mebendazole after collection of samples for primary endpoints at week 8 and 28, and after collection of samples for secondary endpoints at week 52.

Current malaria infection status and intensity will be assessed retrospectively by PCR on stored samples collected on immunisation days and at week 52.

Malarial fever: Individuals presenting with fever will be investigated using rapid diagnostic tests for malaria and treated based on the results and according to prevailing national guidelines.

Prior malaria exposure will be evaluated by ELISA for IgG to malaria antigen using stored samples collected at baseline.

HIV serology will be done on blood samples using rapid tests and according to prevailing national algorithms. The current algorithm is shown in Appendix 2. This will be done at baseline.

Pregnancy testing will be done using urine samples and standard operating procedures for assessment of urine [illegible]-human chorionic gonadotropin[illegible]hCG). This will be done at baseline and before immunisation on each immunisation day.

Full blood counts will be conducted using a haematology analyser. Mild, moderate and severe anaemia will be defined according to WHO guidelines, by age.[14] This will be done at baseline (to test for anaemia as part of the eligibility assessment), and pre-immunisation as part of the assessment of immunological profile.

Individuals found to be HIV positive or pregnant will be referred to appropriate providers for further care.

Individuals with severe anaemia (haemoglobin <82g/L) will be excluded from the randomised intervention (since the intervention might be beneficial in management of anaemia). They will be treated for anaemia and for any underlying cause identified.

Operational considerations

Programme governance

A Programme Steering Committee will be set up to guide progress across all projects. This will comprise the following:

For peer review only

For peer review only - http://bmjopen.bmj.com/site/about/guidelines.xhtml

- An independent chair
- Representatives from the Ministry of Health programmes for immunisation and for vector borne disease control
- Representatives of district authorities (Mukono and Jinja districts)
- Community representatives
- Principal investigator and co-investigators
- Project leader and post-doctoral immunologist
- Trial statistician
- Laboratory manager
- Medical Research Council observer

Informed consent

Both written informed assent from the participants and written informed consent from a parent or guardian will be required for participation, although these may not necessarily be obtained at the same time. Information will be provided in both English and the appropriate local language. For individuals who cannot speak the languages used, or who cannot read or write, a witness who can read the information sheet and translate the information to the participant or parent/guardian will be used. Informed consent by emancipated or mature minors will be obtained using designated consent form for these kinds of participants.

The aims of the study, all tests, treatments and immunisations to be carried out and potential risks will be explained. The participant will be given the opportunity to ask about details of the trial, and will then have time to consider whether or not to participate. If they do decide to participate, they and their parent/guardian will sign and date two copies of the assent and consent forms, one for them to take away and keep, and one to be stored securely by the research team. Separate information and consent forms will be provided for consent for storage of samples for future studies and for anonymous sharing of data from this study. For the EMaBS cohort genetic data are already available based on previous approval; the information sheet will explain that these data may be used in analyses related to this protocol.

Screening and Eligibility Assessment

Once the informed consent process has been completed, and consent (and assent) given, a baseline medical history (including concomitant medication) will be collected. Vital signs will be checked and a physical examination will be performed. Inclusion and exclusion criteria will be checked.

For peer review only

Participants will undergo pre- and post-test counselling for HIV and (for girls) pregnancy testing by a trained and experienced nurse- or clinician-counsellor. Blood, urine and stool samples will be obtained, for tests as specified in the schedule of procedures (Appendices A-C). These tests are to exclude the major, immunomodulating co-infection, HIV, and conditions that might impact safety (anaemia, pregnancy).

Enrolment

Participants who consent/assent, complete the screening processes, satisfy all the inclusion criteria and meet none of the exclusion criteria will be enrolled into the trial. On the enrolment day (which may be the same as the screening day in some cases) eligibility will be checked and participants will be enrolled sequentially to the next randomisation number. They will then be given BCG vaccine or not, according to their allocation.

Discontinuation / withdrawal criteria

In accordance with the principles of the current revision of the Declaration of Helsinki and any other applicable regulations, a participant has the right to withdraw from the study at any time and for any reason, and is not obliged to give his or her reasons for doing so. The Investigator may withdraw the participant at any time in the interests of the participant's health and well-being. In addition, the participant may withdraw/be withdrawn for any of the following reasons:

- Ineligibility (either arising during the study or retrospectively, having been overlooked at screening)
- Administrative decision by the Investigator
- Significant protocol deviation
- Participant non-compliance with study requirements
- An adverse event which requires discontinuation of the study involvement or results in inability to continue to comply with study procedures.

Any participant who becomes pregnant during the trial will be followed up until the end of the pregnancy but no further immunisations will be given unless indicated during pregnancy (as is the case for tetanus toxoid). The trial allocation for this participant will be unblinded and the participant will only be given further treatment if clinically indicated. The babies will also be followed up and examined for any adverse effects. We will not routinely perform venepuncture in a pregnant participant.

For peer review only

The reason for withdrawal will be recorded in the case report form (CRF). If withdrawal is due to an AE, appropriate follow-up visits or medical care will be arranged, with the agreement of the participant, until the AE has resolved, stabilised or a non-trial related causality has been assigned.

If a participant withdraws from the study samples collected before their withdrawal from the trial will be used/ stored unless the participant specifically requests otherwise.

Trial discontinuation

The Trial will be discontinued in the event of new scientific information that renders continuation futile or unethical, or for any other reason, at the discretion of the Programme Steering Committee.

End of study definition

The trial will be completed when the last participant enrolled into the trial has completed their final follow up visit.

Safety assessments and oversight

No new investigational drug or product will be used in the proposed trial. However, standard approaches for monitoring safety and reporting of serious adverse events will be followed.

Monitoring

The trial will be monitored by both internal and external monitors according to a pre-defined monitoring plan which will include a site initiation visit, monitoring visits at least annually, and a close-out visit. The monitors will assess patient safety, data integrity, and adherence to the protocol and to Good Clinical Research Practice procedures.

Procedures to be followed in the event of abnormal findings

Abnormal clinical findings from medical history, examination or blood tests will be assessed as to their clinical significance throughout the trials. If an abnormal test result is deemed clinically significant, it may be repeated. If a test remains clinically significant, the participant will be informed and appropriate medical care arranged as appropriate and with the permission of the participant. Specific details regarding findings, discussion with participants and resulting actions will be recorded in the clinical records. Decisions to exclude the participant from enrolling in the trial or to withdraw a participant from the trial will be at the discretion of the Investigator.

For peer review only

Data and Safety Monitoring Board (DSMB)

A data and safety monitoring board (DSMB) will be appointed to provide real-time safety oversight. The DSMB will be notified within 7 days of the Investigators' being aware of the occurrence of SAEs. The DSMB may recommend the Investigators to place the trial on hold if deemed necessary following an intervention-related SAE. The DSMB will be chaired by a clinician experienced in clinical trials. There will be a minimum of two other appropriately qualified committee members. In the case of events related to a blinded intervention, the DSMB can request unblinding. Membership will include a statistician, and at least one Ugandan member. All correspondence between Investigators and the DSMB will be conveyed by the Principal Investigator to the trial Sponsor. The Chair of the DSMB will be contacted for advice and independent review by the Investigator or trial Sponsor in the following situations:

- The occurrence of any SAE
- Any other situation where the Investigator or trial Sponsor feels independent advice or review is important.

Ethical and regulatory considerations

Information regarding risks and benefits to the participant

Participants in this programme will be adolescents and therefore a vulnerable human population. Care will be taken to provide adequate, age and education-status appropriate information and to ensure that it is understood; and to emphasise that participation is voluntary. Participants will be enrolled only when they have given their own assent and when consent has been given by the parent or guardian.

No major risks to the participants are anticipated since all the treatments and vaccines to be given are licensed and known to be safe. The main risk to participants will be time lost from school work: we will work with parents to minimise disruption to studies.

Participants will suffer the discomfort and inconvenience of providing blood samples (and stool and urine samples). Occasionally people faint when a vaccine is given or when blood is drawn. Individuals will be comfortably seated during these procedures and the research team will be trained to manage such events.

The immunisations to be given have recognised side effects which are usually mild and resolve spontaneously in a few days to one week. Parenteral vaccines are likely to result in pain and swelling at the site of injection and mild fever; very occasionally pain and swelling can be severe and associated with difficulty in moving the shoulder. Sometimes headache and tiredness occurs. Rarely

For peer review only

a vaccine may cause a severe allergic reaction. For most vaccines this is estimated at less than one in a million doses (but 1 in 55,000 for Yellow Fever vaccine).[15] Individuals with a history of a possible allergic reaction to drugs or vaccines, or to vaccine components including eggs or chicken proteins, will be excluded from the studies. The research team will be trained and prepared to manage severe allergic reactions.

Adverse reactions to Yellow Fever vaccine include severe nervous system reaction (about 1 person in 125,000) and severe, life-threatening illness with organ failure (about 1 person in 250,000). The mortality for this severe, life-threatening adverse effect is reported as about 50%.[15]

BCG immunisation is likely to induce a scar in many cases. This may develop over several weeks, starting as a small papule at the injection site which may become ulcerated and then heal over a period of 2 to 5 months; and lymphadenopathy may develop. Occasionally a more severe local reaction occurs (estimated at 1 per 1,000-10,000 doses): for example, an abscess develops and scars may develop into keloids. Rarely BCG can cause disseminated disease (1 per 230,000 to 640,000 doses), or disease in sites remote from the immunisation site. Disseminated BCG disease usually occurs in immunocompromised people: HIV positive people will be excluded from these studies.[16] BCG "pre-immunisation" may interfere with the response to the subsequent live vaccines; indeed our hypothesis, and published results, suggest that it may suppress replication of YF 17D vaccine.[17] However, this reduced replication has not been shown to correlate with, or result in, reduced levels of neutralising antibody titres (which are the desired protective outcome).[4 17]

Oral typhoid vaccine (Ty21a) may occasionally be associated with stomach pain, nausea, vomiting and (rarely) rash.[15]

Benefits

All the vaccines to be given are licensed and regarded as safe. In general, the vaccines and treatments are expected to provide protection against infectious diseases. Participants and their families, and communities are expected to benefit from improved understanding of vaccines.

For peer review only - http://bmjopen.bmj.com/site/about/guidelines.xhtml

References

1. Wajja A, Kizito D, Nassanga B, et al. The effect of current Schistosoma mansoni infection on the immunogenicity of a candidate TB vaccine, MVA85A, in BCG-vaccinated adolescents: An open-label trial. *PLoS Negl Trop Dis* 2017;11(5):e0005440. doi: 10.1371/journal.pntd.0005440 [published Online First: 2017/05/05]

2. WHO. Growth reference 5-19 years. 2007

3. Kraemer MU, Faria NR, Reiner RC, Jr., et al. Spread of yellow fever virus outbreak in Angola and the Democratic Republic of the Congo 2015-16: a modelling study. *The Lancet Infectious diseases* 2016 doi: 10.1016/s1473-3099(16)30513-8 [published Online First: 2016/12/27]

4. Muyanja E, Ssemaganda A, Ngauv P, et al. Immune activation alters cellular and humoral responses to yellow fever 17D vaccine. *The Journal of clinical investigation* 2014;124(7):3147-58. doi: 10.1172/jci75429 [published Online First: 2014/06/10]

5. Aguiar M, Stollenwerk N, Halstead SB. The Impact of the Newly Licensed Dengue Vaccine in Endemic Countries. *PLoS neglected tropical diseases* 2016;10(12):e0005179. doi: 10.1371/journal.pntd.0005179 [published Online First: 2016/12/22]

6. Dharmasena MN, Osorio M, Filipova S, et al. Stable expression of Shigella dysenteriae serotype 1 O-antigen genes integrated into the chromosome of live Salmonella oral vaccine vector Ty21a. *Pathogens and disease* 2016 doi: 10.1093/femspd/ftw098 [published Online First: 2016/09/23]

7. Carias C, Walters MS, Wefula E, et al. Economic evaluation of typhoid vaccination in a prolonged typhoid outbreak setting: the case of Kasese district in Uganda. *Vaccine* 2015;33(17):2079-85. doi: 10.1016/j.vaccine.2015.02.027 [published Online First: 2015/02/26]

8. WHO. Position Paper on Typhoid vaccines: WHO position paper – March 2018 2018

9. Centre HI. HPV and related diseases report: Uganda. 2016. http://www.hpvcentre.net/statistics/reports/UGA.pdf (accessed 20.01.2017).

10. WHO. Human papillomavirus vaccines: WHO position paper, May 2017. *Releve epidemiologique hebdomadaire* 2017;92(19):241-68. [published Online First: 2017/05/23]

11. Nanteza B, Galukande M, Aceng J, et al. The burden of tetanus in Uganda. *SpringerPlus* 2016;5(1):705. doi: 10.1186/s40064-016-2309-z [published Online First: 2016/06/29]

12. Corstjens PL, Nyakundi RK, de Dood CJ, et al. Improved sensitivity of the urine CAA lateral-flow assay for diagnosing active Schistosoma infections by using larger sample volumes. *Parasites & vectors* 2015;8:241. doi: 10.1186/s13071-015-0857-7 [published Online First: 2015/04/22]

13. Webb EL, Nampijja M, Kaweesa J, et al. Helminths are positively associated with atopy and wheeze in Ugandan fishing communities: results from a cross-sectional survey. *Allergy* 2016 doi: 10.1111/all.12867

14. WHO. Haemoglobin concentrations for the diagnosis of anaemia and assessment of severity. Vitamin and Mineral Nutrition Information System. Geneva, World Health Organization, 2011 (WHO/NMH/NHD/MNM/11.1).

15. CDC. Centers for Disease Control and Prevention, vaccines and immunizations.

16. WHO. Information sheet observed rate of vaccine reactions Bacille Calmette-Guérin (BCG) vaccine. 2012

For peer review only

For peer review only - http://bmjopen.bmj.com/site/about/guidelines.xhtml

17. Arts RJW, Moorlag S, Novakovic B, et al. BCG Vaccination Protects against Experimental Viral Infection in Humans through the Induction of Cytokines Associated with Trained Immunity. *Cell host & microbe* 2018;23(1):89-100.e5. doi: 10.1016/j.chom.2017.12.010 [published Online First: 2018/01/13]

For peer review only

For peer review only - http://bmjopen.bmj.com/site/about/guidelines.xhtml

Reporting checklist for protocol of a clinical trial.

Based on the SPIRIT guidelines.

Instructions to authors

Complete this checklist by entering the page numbers from your manuscript where readers will find each of the items listed below.

Your article may not currently address all the items on the checklist. Please modify your text to include the missing information. If you are certain that an item does not apply, please write "n/a" and provide a short explanation.

Upload your completed checklist as an extra file when you submit to a journal.

In your methods section, say that you used the SPIRITreporting guidelines, and cite them as:

Chan A-W, Tetzlaff JM, Altman DG, Laupacis A, Gøtzsche PC, Krleža-Jerić K, Hróbjartsson A, Mann H, Dickersin K, Berlin J, Doré C, Parulekar W, Summerskill W, Groves T, Schulz K, Sox H, Rockhold FW, Rennie D, Moher D. SPIRIT 2013 Statement: Defining standard protocol items for clinical trials. Ann Intern Med. 2013;158(3):200-207

		Reporting Item	Page Number
**Administrative information**			
Title	#1	Descriptive title identifying the study design, population, interventions, and, if applicable, trial acronym	1
Trial registration	#2a	Trial identifier and registry name. If not yet registered, name of intended registry	2
Trial registration: data set	#2b	All items from the World Health Organization Trial Registration Data Set	n/a
Protocol version	#3	Date and version identifier	Information available at ISRCTN10482904
Funding	#4	Sources and types of financial, material, and other support	15

For peer review only

Roles and responsibilities: contributorship	#5a	Names, affiliations, and roles of protocol contributors	15
Roles and responsibilities: sponsor contact information	#5b	Name and contact information for the trial sponsor	Information avaioalable at ISRCTN10482904
Roles and responsibilities: sponsor and funder	#5c	Role of study sponsor and funders, if any, in study design; collection, management, analysis, and interpretation of data; writing of the report; and the decision to submit the report for publication, including whether they will have ultimate authority over any of these activities	16
Roles and responsibilities: committees	#5d	Composition, roles, and responsibilities of the coordinating centre, steering committee, endpoint adjudication committee, data management team, and other individuals or groups overseeing the trial, if applicable (see Item 21a for data monitoring committee)	Supplementary information – Pg. 6 and 10
**Introduction**			
Background and rationale	#6a	Description of research question and justification for undertaking the trial, including summary of relevant studies (published and unpublished) examining benefits and harms for each intervention	4 and 5
Background and rationale: choice of comparators	#6b	Explanation for choice of comparators	7
Objectives	#7	Specific objectives or hypotheses	6
Trial design	#8	Description of trial design including type of trial (eg, parallel group, crossover, factorial, single group), allocation ratio, and framework (eg, superiority, equivalence, non-inferiority, exploratory)	7

For peer review only

**Methods: Participants, interventions, and outcomes**			
Study setting	#9	Description of study settings (eg, community clinic, academic hospital) and list of countries where data will be collected. Reference to where list of study sites can be obtained	6
Eligibility criteria	#10	Inclusion and exclusion criteria for participants. If applicable, eligibility criteria for study centres and individuals who will perform the interventions (eg, surgeons, psychotherapists)	6 and 7
Interventions: description	#11a	Interventions for each group with sufficient detail to allow replication, including how and when they will be administered	8
Interventions: modifications	#11b	Criteria for discontinuing or modifying allocated interventions for a given trial participant (eg, drug dose change in response to harms, participant request, or improving / worsening disease)	Supplementary information
Interventions: adherance	#11c	Strategies to improve adherence to intervention protocols, and any procedures for monitoring adherence (eg, drug tablet return; laboratory tests)	Supplementary information
Interventions: concomitant care	#11d	Relevant concomitant care and interventions that are permitted or prohibited during the trial	n/a; participants are not expected to be receiving any concomitant care and interventions during the study
Outcomes	#12	Primary, secondary, and other outcomes, including the specific measurement variable (eg, systolic blood pressure), analysis metric (eg, change from baseline, final value, time to event), method of aggregation (eg, median, proportion), and time point for each outcome. Explanation of the clinical relevance of chosen efficacy and harm outcomes is strongly recommended	10

For peer review only

Participant timeline	#13	Time schedule of enrolment, interventions (including any run-ins and washouts), assessments, and visits for participants. A schematic diagram is highly recommended (see Figure)	Supplementary information; Table S1, pg 2
Sample size	#14	Estimated number of participants needed to achieve study objectives and how it was determined, including clinical and statistical assumptions supporting any sample size calculations	11
Recruitment	#15	Strategies for achieving adequate participant enrolment to reach target sample size	12
**Methods: Assignment of interventions (for controlled trials)**			
Allocation: sequence generation	#16a	Method of generating the allocation sequence (eg, computer-generated random numbers), and list of any factors for stratification. To reduce predictability of a random sequence, details of any planned restriction (eg, blocking) should be provided in a separate document that is unavailable to those who enrol participants or assign interventions	8
Allocation concealment mechanism	#16b	Mechanism of implementing the allocation sequence (eg, central telephone; sequentially numbered, opaque, sealed envelopes), describing any steps to conceal the sequence until interventions are assigned	8
Allocation: implementation	#16c	Who will generate the allocation sequence, who will enrol participants, and who will assign participants to interventions	8
Blinding (masking)	#17a	Who will be blinded after assignment to interventions (eg, trial participants, care	8

For peer review only

		providers, outcome assessors, data analysts), and how	
Blinding (masking): emergency unblinding	#17b	If blinded, circumstances under which unblinding is permissible, and procedure for revealing a participant's allocated intervention during the trial	Supplementary information – Pg. 10
**Methods: Data collection, management, and analysis**			
Data collection plan	#18a	Plans for assessment and collection of outcome, baseline, and other trial data, including any related processes to promote data quality (eg, duplicate measurements, training of assessors) and a description of study instruments (eg, questionnaires, laboratory tests) along with their reliability and validity, if known. Reference to where data collection forms can be found, if not in the protocol	12
Data collection plan: retention	#18b	Plans to promote participant retention and complete follow-up, including list of any outcome data to be collected for participants who discontinue or deviate from intervention protocols	12
Data management	#19	Plans for data entry, coding, security, and storage, including any related processes to promote data quality (eg, double data entry; range checks for data values). Reference to where details of data management procedures can be found, if not in the protocol	12.<br><br>These will also be detailed in a statistical analysis plan that will be uploaded to the online trial registration.
Statistics: outcomes	#20a	Statistical methods for analysing primary and secondary outcomes. Reference to where other details of the statistical analysis plan can be found, if not in the protocol	12.<br><br>These will also be detailed in the statistical analysis plan that will be uploaded to the online trial registration.

For peer review only

Statistics: additional analyses	#20b	Methods for any additional analyses (eg, subgroup and adjusted analyses)	12.<br><br>These will also be detailed in the statistical analysis plan that will be uploaded to the online trial registration.
Statistics: analysis population and missing data	#20c	Definition of analysis population relating to protocol non-adherence (eg, as randomised analysis), and any statistical methods to handle missing data (eg, multiple imputation)	These will be detailed in the statistical analysis plan that will be uploaded to the online trial registration.
**Methods: Monitoring**			
Data monitoring: formal committee	#21a	Composition of data monitoring committee (DMC); summary of its role and reporting structure; statement of whether it is independent from the sponsor and competing interests; and reference to where further details about its charter can be found, if not in the protocol. Alternatively, an explanation of why a DMC is not needed	Supplementary information – Pg. 10
Data monitoring: interim analysis	#21b	Description of any interim analyses and stopping guidelines, including who will have access to these interim results and make the final decision to terminate the trial	Supplementary information – Pg. 9
Harms	#22	Plans for collecting, assessing, reporting, and managing solicited and spontaneously reported adverse events and other unintended effects of trial interventions or trial conduct	Supplementary information – Pg. 10
Auditing	#23	Frequency and procedures for auditing trial conduct, if any, and whether the process will be independent from investigators and the sponsor	Supplementary information – Pg. 9
**Ethics and dissemination**			

For peer review only

Research ethics approval	#24	Plans for seeking research ethics committee / institutional review board (REC / IRB) approval	11
Protocol amendments	#25	Plans for communicating important protocol modifications (eg, changes to eligibility criteria, outcomes, analyses) to relevant parties (eg, investigators, REC / IRBs, trial participants, trial registries, journals, regulators)	11
Consent or assent	#26a	Who will obtain informed consent or assent from potential trial participants or authorised surrogates, and how (see Item 32)	12 and Supplementary information – Pg. 7
Consent or assent: ancillary studies	#26b	Additional consent provisions for collection and use of participant data and biological specimens in ancillary studies, if applicable	Supplementary information – Pg. 7
Confidentiality	#27	How personal information about potential and enrolled participants will be collected, shared, and maintained in order to protect confidentiality before, during, and after the trial	12
Declaration of interests	#28	Financial and other competing interests for principal investigators for the overall trial and each study site	15
Data access	#29	Statement of who will have access to the final trial dataset, and disclosure of contractual agreements that limit such access for investigators	n/a
Ancillary and post trial care	#30	Provisions, if any, for ancillary and post-trial care, and for compensation to those who suffer harm from trial participation	Supplementary information – Pg. 10
Dissemination policy: trial results	#31a	Plans for investigators and sponsor to communicate trial results to participants, healthcare professionals, the public, and other relevant groups (eg, via publication, reporting in results databases, or other data sharing arrangements), including any publication restrictions	2, 12

For peer review only

Dissemination policy: authorship	#31b	Authorship eligibility guidelines and any intended use of professional writers	n/a
Dissemination policy: reproducible research	#31c	Plans, if any, for granting public access to the full protocol, participant-level dataset, and statistical code	12

Appendices

Informed consent materials	#32	Model consent form and other related documentation given to participants and authorised surrogates	n/a
Biological specimens	#33	Plans for collection, laboratory evaluation, and storage of biological specimens for genetic or molecular analysis in the current trial and for future use in ancillary studies, if applicable	n/a

None The SPIRIT checklist is distributed under the terms of the Creative Commons Attribution License CC-BY-ND 3.0. This checklist can be completed online using https://www.goodreports.org/, a tool made by the EQUATOR Network in collaboration with Penelope.ai

For peer review only

BMJ Open

The impact of Bacillus Calmette-Guérin revaccination on the response to unrelated vaccines in a Ugandan adolescent birth cohort: randomised controlled trial protocol C for the 'POPulation differences in VACcine responses' (POPVAC) programme

Journal:	*BMJ Open*
Manuscript ID	bmjopen-2020-040430.R1
Article Type:	Protocol
Date Submitted by the Author:	07-Sep-2020

Complete List of Authors:	Zirimenya , Ludoviko ; MRC/UVRI and LSHTM Uganda Research Unit, Immunomodulation and Vaccines Programme<br>Nkurunungi, Gyaviira; MRC/UVRI and LSHTM Uganda Research Unit, Immunomodulation and Vaccines Programme<br>Nassuuna , Jacent; MRC/UVRI and LSHTM Uganda Research Unit, Immunomodulation and Vaccines Programme<br>Natukunda , Agnes ; MRC/UVRI and LSHTM Uganda Research Unit, Immunomodulation and Vaccines Programme<br>Mutebe , Alex ; MRC/UVRI and LSHTM Uganda Research Unit, Immunomodulation and Vaccines Programme<br>Oduru, Gloria; MRC/UVRI and LSHTM Uganda Research Unit, Immunomodulation and Vaccines Programme<br>Kabami , Grace ; MRC/UVRI and LSHTM Uganda Research Unit, Immunomodulation and Vaccines Programme<br>Akurut, Hellen; MRC/UVRI and LSHTM Uganda Research Unit, Immunomodulation and Vaccines Programme<br>Onen, Caroline; MRC/UVRI and LSHTM Uganda Research Unit, Immunomodulation and Vaccines Programme<br>Namutebi , Milly ; MRC/UVRI and LSHTM Uganda Research Unit, Immunomodulation and Vaccines Programme<br>Serubanja, Joel; MRC/UVRI and LSHTM Uganda Research Unit, Immunomodulation and Vaccines Programme<br>Nakazibwe, Esther; MRC/UVRI and LSHTM Uganda Research Unit, Immunomodulation and Vaccines Programme<br>Akello , Florence ; MRC/UVRI and LSHTM Uganda Research Unit, Immunomodulation and Vaccines Programme<br>Tumusiime, Josephine; MRC/UVRI and LSHTM Uganda Research Unit, Immunomodulation and Vaccines Programme<br>Sewankambo, Moses; MRC/UVRI and LSHTM Uganda Research Unit, Immunomodulation and Vaccines Programme<br>Kiwanuka, Samuel; MRC/UVRI and LSHTM Uganda Research Unit, Immunomodulation and Vaccines Programme<br>Kiwudhu, Fred; MRC/UVRI and LSHTM Uganda Research Unit, Immunomodulation and Vaccines Programme<br>Kizindo , Robert ; MRC/UVRI and LSHTM Uganda Research Unit, Immunomodulation and Vaccines Programme<br>Kizza, Moses; MRC/UVRI and LSHTM Uganda Research Unit,

	Immunomodulation and Vaccines Programme<br>Wajja, Anne; MRC/UVRI and LSHTM Uganda Research Unit, Immunomodulation and Vaccines Programme<br>Cose , Stephen ; MRC/UVRI and LSHTM Uganda Research Unit, Immunomodulation and Vaccines Programme; London School of Hygiene and Tropical Medicine Department of Clinical Research<br>Muwanga, Moses; Entebbe Hospital<br>Webb, Emily; London School of Hygiene & Tropical Medicine, MRC Tropical Epidemiology Group, Department of Infectious Disease Epidemiology<br>Elliott, Alison ; MRC/UVRI and LSHTM Uganda Research Unit, Immunomodulation and Vaccines Programme; London School of Hygiene and Tropical Medicine Department of Clinical Research

**Primary Subject Heading**:	Infectious diseases
Secondary Subject Heading:	Immunology (including allergy), Infectious diseases, Epidemiology, Public health
Keywords:	Infection control < INFECTIOUS DISEASES, Public health < INFECTIOUS DISEASES, Immunology < TROPICAL MEDICINE, Epidemiology < TROPICAL MEDICINE, Paediatric infectious disease & immunisation < PAEDIATRICS

SCHOLARONE™
Manuscripts

I, the Submitting Author has the right to grant and does grant on behalf of all authors of the Work (as defined in the below author licence), an exclusive licence and/or a non-exclusive licence for contributions from authors who are: i) UK Crown employees; ii) where BMJ has agreed a CC-BY licence shall apply, and/or iii) in accordance with the terms applicable for US Federal Government officers or employees acting as part of their official duties; on a worldwide, perpetual, irrevocable, royalty-free basis to BMJ Publishing Group Ltd ("BMJ") its licensees and where the relevant Journal is co-owned by BMJ to the co-owners of the Journal, to publish the Work in this journal and any other BMJ products and to exploit all rights, as set out in our licence.

The Submitting Author accepts and understands that any supply made under these terms is made by BMJ to the Submitting Author unless you are acting as an employee on behalf of your employer or a postgraduate student of an affiliated institution which is paying any applicable article publishing charge ("APC") for Open Access articles. Where the Submitting Author wishes to make the Work available on an Open Access basis (and intends to pay the relevant APC), the terms of reuse of such Open Access shall be governed by a Creative Commons licence – details of these licences and which Creative Commons licence will apply to this Work are set out in our licence referred to above.

Other than as permitted in any relevant BMJ Author's Self Archiving Policies, I confirm this Work has not been accepted for publication elsewhere, is not being considered for publication elsewhere and does not duplicate material already published. I confirm all authors consent to publication of this Work and authorise the granting of this licence.

For peer review only

The impact of Bacillus Calmette-Guérin revaccination on the response to unrelated vaccines in a Ugandan adolescent birth cohort: randomised controlled trial protocol C for the 'POPulation differences in VACcine responses' (POPVAC) programme

Ludoviko Zirimenya[1,¶], Gyaviira Nkurunungi[1,¶,*], Jacent Nassuuna[1,¶], Agnes Natukunda[1,¶], Alex Mutebe[1], Gloria Oduru[1], Grace Kabami[1], Hellen Akurut[1], Caroline Onen[1], Milly Namutebi[1], Joel Serubanja[1], Esther Nakazibwe[1], Florence Akello[1], Josephine Tumusiime[1], Moses Sewankambo[1], Samuel Kiwanuka[1], Fred Kiwudhu[1], Robert Kizindo[1], Moses Kizza[1], Anne Wajja[1], Stephen Cose[1,2], Moses Muwanga[3], Emily L Webb[4], Alison M Elliott[1,2] **for the POPVAC trial team**

[1]Immunomodulation and Vaccines Programme, Medical Research Council/Uganda Virus Research Institute and London School of Hygiene and Tropical Medicine (MRC/UVRI and LSHTM) Uganda Research Unit, Entebbe, Uganda

[2]Department of Clinical Research, London School of Hygiene and Tropical Medicine, London, United Kingdom

[3]Entebbe Hospital, Entebbe, Uganda

[4]MRC Tropical Epidemiology Group, Department of Infectious Disease Epidemiology, London School of Hygiene and Tropical Medicine, London, United Kingdom

[¶]These authors contributed equally

***Correspondence:** Gyaviira Nkurunungi; Gyaviira.Nkurunungi@mrcuganda.org

For peer review only

Abstract

Introduction

There is evidence that Bacillus Calmette–Guérin (BCG) immunisation may protect against unrelated infectious illnesses. This has led to the postulation that administering BCG before unrelated vaccines may enhance responses to these vaccines. This might also model effects of BCG on unrelated infections.

Methods and analysis

To test this hypothesis, we have designed a randomised controlled trial of BCG versus no BCG immunisation to determine the effect of BCG on subsequent unrelated vaccines, among 300 adolescents (ages 13 to 17 years) from a Ugandan birth cohort. Our schedule will comprise three main immunisation days (week 0, week 4 and week 28): BCG (or no BCG) revaccination at week 0, Yellow fever (YF-17D), Oral typhoid (Ty21a) and HPV prime at week 4, HPV boost and Tetanus/diphtheria (Td) boost at week 28. Primary outcomes are anti-YF-17D neutralising antibody titres, *Salmonella typhi* lipopolysaccharide (LPS)-specific IgG concentration, IgG specific for L1-proteins of HPV-16/18 and tetanus and diphtheria toxoid-specific IgG concentration, all assessed at four weeks after immunisation with YF, Ty21a, HPV and Td, respectively. Secondary analyses will determine effects on correlates of protective immunity (where recognised correlates exist), on vaccine response waning and on whether there are differential effects on priming vs boosting immunisations. We will also conduct exploratory immunology assays among subsets of participants to further characterise effects of BCG revaccination on vaccine responses. Further analyses will assess which life-course exposures influence vaccine responses in adolescence.

Ethics and dissemination

Ethics approval has been obtained from relevant Ugandan and UK ethics committees. Results will be shared with Uganda Ministry of Health, relevant district councils, community leaders and study participants. Further dissemination will be done through conference proceedings and publications.

Trial registration

Current Controlled Trials identifier: ISRCTN10482904

Article summary

Strengths and limitations of this study

For peer review only

For peer review only - http://bmjopen.bmj.com/site/about/guidelines.xhtml

- This will be the first well-powered trial to investigate effects of BCG revaccination on responses to unrelated vaccines in adolescents.
- Effects on both live-attenuated and inert vaccines will be studied.
- Our robust immunoepidemiological design and nested immunological studies will address specific hypotheses regarding pathways of effects of BCG immunisation on unrelated vaccine responses.
- One limitation is that interaction between the three vaccines administered together one month after BCG immunisation may mask the true effect of BCG revaccination on individual vaccine responses.

Word count

3005

Keywords

Vaccine; BCG; Immunization; Uganda

For peer review only

For peer review only - http://bmjopen.bmj.com/site/about/guidelines.xhtml

Introduction

There is increasing evidence that Bacillus Calmette–Guérin (BCG) immunisation has non-specific, protective effects relating to infections other than tuberculosis.[1-4] Experimental studies using BCG suggest that effects on the innate immune response are an important component of this phenomenon: BCG immunisation induces lasting epigenetic modification of innate immune cells, including monocytes, macrophages and natural killer cells.[5-8] This process, by which the innate immune system develops a form of memory, has been called "trained innate immunity".[9] Evidence is accumulating that a range of stimuli including bacterial products (particularly *Salmonella typhi* lipopolysaccharide (LPS)), and infections including malaria and hepatitis B,[10] may induce trained innate immunity; that the profile into which cells are trained varies with the dose and characteristics of the stimulus; and that effects may be induced prenatally (on exposure to maternal infections) as well as later in life.[9]

It is plausible that variation in the intensity and spectrum of experience of previous infections, and hence the epigenetic programming and consequent functional profiles of innate immune cells, contributes to the many differences in immunological activity observed between geographically and environmentally distinct settings, and hence to differences in vaccine response. If this hypothesis is correct, BCG immunisation can act as a model for the effects of prior infection, and may also be a tool for inducing enhanced benefits for other vaccines. Vaccine-specific responses can also act as a model for responses to infection. This is especially relevant given the current interest in the potential benefit of BCG immunisation against COVID-19 disease.[11 12]

In Europe, BCG vaccination two weeks before giving influenza vaccine has been shown to result in enhanced antibody responses to influenza proteins.[13] BCG immunisation four weeks before giving Yellow Fever (YF 17D) vaccine has also been found to result in reduced replication of the yellow fever vaccine virus; this was not associated with a significant reduction in the desired neutralising antibody response to YF, or in the interferon (IFN)-γ response, but the study size was small and may not have had sufficient power to demonstrate important effects.[14]

In Uganda, BCG immunisation at birth is recommended.[15] The benefits of BCG immunisation in adolescence for protection against tuberculosis are not known and may differ between settings.[16] Whether BCG immunisation in adolescents in Uganda will have non-specific effects on the innate immune response, on subsequent immunisations and (indeed) on general health (given the prior exposure at birth, and the on-going exposure to non-tuberculous mycobacteria and other infections) is not known. In Protocol C of the 'Population differences in Vaccine responses" programme

For peer review only - http://bmjopen.bmj.com/site/about/guidelines.xhtml

96 (POPVAC C; Current Controlled Trials identifier: ISRCTN10482904), we plan to address this knowledge gap by randomising adolescent members of the Entebbe Mother and Baby Study (EMaBS) birth cohort[15] in a nested trial of BCG revaccination versus no BCG revaccination prior to immunisation with other vaccines. We summarise the protocol here.

For peer review only

For peer review only - http://bmjopen.bmj.com/site/about/guidelines.xhtml

Hypothesis

The overarching goal of the POPVAC programme is to understand population differences in vaccine responses in Uganda, in order to identify strategies through which vaccine effectiveness can be optimised for the low-income, tropical settings where they are especially needed. For this Trial C we address the concept of trained innate immunity through the hypothesis that BCG immunisation modifies the response to subsequent unrelated vaccines.

Objective

To determine whether BCG revaccination modulates the response to unrelated vaccines among Ugandan adolescents.

For peer review only

For peer review only - http://bmjopen.bmj.com/site/about/guidelines.xhtml

Methods and analysis

Setting and participants

SPIRIT reporting guidelines[17] are used. This trial will be a randomised, controlled, open, parallel group trial investigating the effect of BCG revaccination on unrelated vaccine response outcomes. The study will take place in Entebbe municipality, Wakiso District, Uganda and will involve participants in the EMaBS birth cohort.[15] In EMaBS, a cohort of 2500 pregnant women were recruited between 2003 and 2005 for a trial of anthelminthic treatment during pregnancy and early childhood, investigating effects on childhood vaccine responses and infectious disease incidence.[15] We aim to enroll 300 of the EMaBS birth cohort participants, randomising 150 to each intervention arm. All EMaBS participants received BCG at birth; hence current trial participants (in the BCG intervention arm) will undergo revaccination. EMaBS participants are expected to be aged 13 to 17 during recruitment to this study. As part of the on-going cohort follow-up, participants will be encouraged to attend the clinic for interim illness events and all serious adverse events, including hospitalisations, will be documented.

Recruitment criteria

Inclusion criteria

i. A participant in the Entebbe Mother and Baby Study[15]
ii. Written informed consent by parent or guardian
iii. Written informed assent by participant
iv. Willing to remain in the study area for the duration of the study
v. Willing to provide locator information and to be contacted during the course of the trial
vi. Females agree to avoid pregnancy for the duration of the trial
vii. Able and willing (in the investigator's opinion) to comply with all the study requirements

Exclusion criteria

i. Concurrent enrolment into another clinical trial
ii. Clinically significant history of immunodeficiency (including HIV), cancer, cardiovascular disease, gastrointestinal disease, liver disease, renal disease, endocrine disorder and neurological illness
iii. History of serious psychiatric condition or disorder
iv. Moderate or severe acute illness characterised by any of the following symptoms: fever, impaired consciousness, convulsions, difficulty in breathing, vomiting; or as determined by the attending project clinician.

For peer review only

v. History of previous immunisation with Yellow Fever (YF), oral typhoid or Human Papillomavirus (HPV) vaccine; previous immunisation with BCG or Tetanus and diphtheria vaccine (Td) at age ≥5 years

vi. Concurrent oral or systemic steroid medication or the concurrent use of other immunosuppressive agents within 2 months prior to enrolment

vii. History of allergic reaction to immunisation or any allergy likely to be exacerbated by any component of the study vaccines including egg or chicken proteins

viii. Tendency to develop keloid scars

ix. Positive HIV serology

x. Positive pregnancy test

xi. Female currently lactating, confirmed pregnancy or intention to become pregnant during the trial period

xii. Use of an investigational medicinal product or non-registered drug, live vaccine, or medical device other than the study vaccines for 30 days prior to dosing with the study vaccine, or planned use during the study period

xiii. Administration of immunoglobulins and/or any blood products within the three months preceding the planned trial immunisation date

Interventions

We will randomise participants to receive BCG or not to receive BCG, four weeks prior to immunisation with a panel of licensed unrelated vaccines (discussed below). The adolescents in the intervention arm will receive a dose of 0.1 ml of BCG-Russia (Serum Institute of India) in the deltoid region of the right upper arm.

Randomisation and allocation to treatment arm

An independent statistician will generate the randomisation code using a randomly permuted block size. This code will be embedded as a web-based randomisation system in REDCap (Research Electronic Data Capture) software.[18,19] Randomisation to the two trial arms will be done in a 1:1 ratio. At enrolment, eligibility criteria will be checked and eligible participants will be allocated sequentially to the next randomisation number, with the corresponding trial arm designated in REDCap. The randomisation code will be kept securely by the trial statistician with a second copy held by a data manager or statistician not otherwise involved in the trial at the MRC/UVRI and LSHTM Uganda Research Unit.

For peer review only

Blinding

This trial will not be blinded to clinicians or participants since they will not participate in outcome ascertainment and the expected development of a BCG skin reaction makes blinding difficult. It is unlikely that participants allocated to "no BCG" will seek this privately. Only laboratory personnel evaluating vaccine response outcomes will be unaware of BCG allocation so outcome ascertainment will not be biased through lack of blinding.

Immunisations

We anticipate that BCG revaccination may have different effects on live and non-live, oral and parenteral, priming and boosting vaccines. Activated innate responses may kill live vaccines and suppress subsequent adaptive responses by this, or other, mechanisms,[20 21] but bias, or even enhance, responses to toxoids or proteins;[22-24] thus, results from a single-vaccine study would not be generalisable.

We therefore propose to study a portfolio of licensed vaccines (live and inert, oral and parental, priming and boosting) expected to be beneficial (in some cases, already given) to adolescents in Uganda. Our schedule (**Table 1** and supplementary **Table S1**) will comprise three main immunisation days (week 0, week 4 and week 28). Additional HPV immunisation will be provided for girls aged 14 years or above, and a second Td boost will be given after completion of the study, to accord with the national Expanded Programme on Immunisation (EPI) routines but the response to these will not specifically be addressed. Further rationale for the selection of vaccines is detailed in supplementary information. Our schedule has been developed in consultation with the EPI programme and is cognizant of potential interference between vaccines.

Table 1. Immunisation schedule

	**Immunisation week 0**	**Immunisation week 4**	**[Immunisation week 8]**	**Immunisation week 28**	**[Immunisation week 52]**
**Live vaccines**	BCG re-vaccination[1]	Yellow fever (YF-17D)<br>Oral typhoid (Ty21a)			
**Non-live vaccines**		HPV prime	HPV boost for girls aged ≥14 years[2,3]	HPV boost and Tetanus/ diphtheria (Td) boost	Tetanus/ diphtheria (Td) boost[3,4]

1. Prior BCG status may vary (data on history and documentation of prior BCG, and presence of a BCG scar, will be documented although these approaches have limitations for determining BCG status)
2. The National EPI programme recommends three doses of HPV vaccine for older girls
3. These doses will be given to comply with guidelines but outcomes specifically relating to these doses will not be assessed
4. Priming by immunisation in infancy is assumed

For peer review only

Schedule of immunisation and sampling

The schedule of immunisation and sampling is outlined in **Table S1**. While optimal timings for outcome measures vary between vaccines, sampling at 8 weeks post BCG and 4 weeks post YF-17D, Ty21a, HPV and Td is proposed for the primary endpoints, targeting the establishment of memory responses and approximate peak of antibody responses. A secondary endpoint at one year will assess waning. All analyses will take baseline measurements into account. Immunisation postponement criteria are detailed in Supplementary information.

Outcomes

Primary outcomes

These will be assessed in all participants.

i. **YF-17D**: neutralising antibody titres (plaque-reduction neutralisation test) at four weeks post YF immunisation.
ii. **Ty21a**: *Salmonella typhi* lipopolysaccharide (LPS)-specific immunoglobulin (Ig)G concentration at four weeks post Ty21a immunisation.
iii. **HPV:** IgG specific for L1-proteins of HPV-16/18 at four weeks post HPV priming immunisation.
iv. **Td:** tetanus and diphtheria toxoid-specific IgG concentration at four weeks post Td immunisation.

Secondary outcomes

These will be assessed in all participants and will further investigate estimates of protective immunity (for vaccines where these are available) and dynamics of the vaccine responses, as well as the impact of the interventions on parasite clearance.

i. **Protective immunity**: Proportions with protective neutralising antibody (YF); protective IgG levels (TT);[25] seroconversion rates (Ty21a) at four weeks post the corresponding immunisation.
ii. **Response waning**: Primary outcome measures (all vaccines) repeated at week 52, and area-under-the curve (AUC) analyses. Parasitic infection may accelerate,[26] and anti-parasitic interventions delay, waning.
iii. **Priming versus boosting**: Effects on priming versus boosting will be examined for HPV only, comparing outcomes four weeks after the first, and four weeks after the second vaccine dose.

For peer review only

Furthermore, our sample collection will offer opportunities for an array of exploratory immunological evaluations on stored samples, focusing mainly on vaccine antigen specific outcomes. Exploratory assays will provide further detail on the mechanisms underlying effects of BCG on responses to unrelated vaccines. Such assays will assess the effects of revaccination with BCG on the profile of cellular phenotypes established prior to immunisation with the later-scheduled vaccines. For example, samples collected will provide opportunities for profiling using mass and flow cytometry, markers of immune activation and regulation, and gene expression studies.

Additional measurements

Other additional assays are discussed in Supplementary information, and will comprise evaluation of helminth and malaria infection exposure, HIV serology (at baseline), pregnancy and full blood count testing (at baseline and before immunisation on each immunisation day).

Sample size considerations

Based on the literature[20 27 28] and preliminary data, we anticipate that standard deviations (SDs) of primary outcome measures will lie between 0.3 and 0.6 $\log_{10}$; and that revaccination with BCG may increase responses by approximately 0.12-0.14 $\log_{10}$. Based on these assumptions, we aim to enrol 300 EMaBS participants (150 BCG revaccination, 150 no BCG revaccination). Allowing for 10% loss to follow-up, this will give over 90% power to detect a difference of 0.12$\log_{10}$ in vaccine response between the pre-BCG immunised and non-pre-immunised groups, at 5% significance level and assuming vaccine response standard deviation of 0.3$\log_{10}$ (**Table 2**).

Table 2. Power estimates (5% significance level)

Standard deviation ($\log_{10}$)	$\log_{10}$ difference						
	**0.08**	**0.10**	**0.12**	**0.14**	**0.16**	**0.18**	**0.20**
Trial C: 150 BCG immunisation vs 150 no BCG immunisation							
0.3	59%	78%	91%	97%	99%	>99%	>99%
0.4	37%	53%	69%	82%	91%	96%	98%
0.5	26%	37%	50%	63%	75%	84%	91%
0.6	19%	28%	37%	48%	59%	69%	78%

Cells highlighted in grey correspond to >80% power.

Ethics and Dissemination

Ethical approval has been granted from the Research Ethics Committees of the Uganda Virus Research Institute (reference: GC/127/19/05/682), the London School of Hygiene and Tropical Medicine (reference: 16034), the Uganda National Council for Science and Technology (reference: HS 2491) and from the Uganda National Drug Authority (certificate number: CTA0094). Any protocol

For peer review only - http://bmjopen.bmj.com/site/about/guidelines.xhtml

For peer review only

amendments will be submitted to ethics committees and regulatory bodies for approval before implementation.

Participants will be adolescents and therefore a vulnerable human population. Care will be taken to provide adequate, age- and education-status appropriate information and to ensure that it is understood; and to emphasise that participation is voluntary. Participants will be enrolled only when they have given their own assent and when consent has been given by the parent or guardian. No major risks to the participants are anticipated since all the vaccines to be given are licensed and known to be safe.

With regard to BCG immunisation or revaccination in adolescence, benefits with respect to protection against tuberculosis among Ugandan adolescents are unknown and may, at best, be modest. There may be non-specific benefits. WHO's SAGE committee concluded, in their summary of October 2017,[29] that "BCG revaccination is safe in *Mycobacterium tuberculosis* infected and uninfected populations. There is a lack of evidence from randomised controlled trials and retrospective cohort and case-control studies demonstrating the efficacy and effectiveness of BCG revaccination in adolescents and adults after primary BCG vaccination in infancy for protection against TB disease. Due to absence of evidence, BCG revaccination is not considered cost-effective. Further research is warranted to explore whether certain sub-groups of age, geographic or *M. tuberculosis* exposure categories would benefit from BCG revaccination." We hope, through this work, to contribute to this debate.

Study findings will be published through open access peer-reviewed journals, presentations at local, national and international conferences and to the local community through community meetings. Anonymised participant level datasets generated will be available upon request.

Patient and public involvement

The EMaBS research team has previously worked with volunteer local council field workers to ensure regular follow up of participants and these field workers continue to attend participants' meetings and provide a mechanism by which the communities from which participants are drawn can be informed about on-going work. As well, prior to the start of this study, we will share our plans with district health and education officers, and with colleagues at Entebbe Hospital. We will establish an advisory committee of parents who will help us to ensure that EMaBS cohort members can participate in the study without undue disruption to their school work. Study findings will be shared with these stakeholders and with participants.

For peer review only - http://bmjopen.bmj.com/site/about/guidelines.xhtml

Data management and analysis

Socio-demographic information and clinical and laboratory measurements will be recorded and managed using REDCap (Research Electronic Data Capture) tools,[18 19] with paper-based forms as back-up. All data will be recorded under a unique study ID number. When paper forms must be used, data will be double entered in a study-specific database, with standard checks for discrepancies. All data for analysis will be anonymised and stored on a secure and password-protected server, with access limited to essential research personnel.

The effect of BCG versus no BCG revaccination on the outcomes will be analysed, including sub group analysis by sex. The analysis will test whether BCG pre-immunisation alters the response to live or inert vaccines given four weeks later, including effects on vaccine replication, immune response profile, priming, boosting and waning. It will indicate whether including BCG as a component of school-based immunisation schedules is likely to have non-specific benefits for Ugandan adolescents.

For peer review only

Discussion

It is increasingly clear that several live vaccines, including BCG, measles vaccine and Vaccinia (smallpox) vaccine, have non-specific, beneficial, effects including reduced mortality (not related to the infectious disease that they were designed to target).[1 2] The potential effects of BCG on responses to unrelated vaccines, specifically on live-attenuated ones such as yellow fever and oral typhoid, might model its effects on responses to unrelated infectious agents.

In contrast, non-specific negative effects have been associated with inactivated vaccines such as diphtheria-tetanus-pertussis (DTP). A high childhood mortality has been observed among girls vaccinated with DTP.[30 31] It has been further suggested that reducing time of exposure to DTP as the most recent vaccination with BCG may reduce this childhood mortality. [30]

We hypothesise that BCG immunisation both achieves non-specific benefits, and influences vaccine responses, through mechanisms based on effects on the innate immune system and consequent immunological profile.

Of note, in this Ugandan birth cohort, all participants were documented to have received BCG at birth, with the strain of BCG used recorded.[15] This will therefore be the first well-powered study to investigate effects of BCG revaccination on vaccine responses in adolescents. It will not investigate the effects of a first dose of BCG in adolescence.

For this work, all participants will receive BCG-Russia strain, provided by the Serum Institute of India. While responses to strains vary, this strain is widely available globally, and in use in Uganda. For comparability, it will be used across the three trials, POPVAC A, B and C. In the context of these trials it will not be possible to determine whether different strains of BCG would have different effects on other vaccines.

This study will determine whether BCG immunisation alters the response to live or inert vaccines given four weeks later, including effects on vaccine replication, immune response profile, priming, boosting and waning among adolescents who received BCG as infants. It will indicate whether including BCG as a component of school-based immunisation schedules is likely to have non-specific benefits for Ugandan adolescents and other settings where infant BCG immunisation is common. If this is correct, BCG immunisation may be used as a tool for inducing enhanced benefits for other vaccines in a wide range of settings.

Study timeline

For peer review only

For peer review only - http://bmjopen.bmj.com/site/about/guidelines.xhtml

Applications for ethical approval were submitted in May 2018, with approval received in September 2018 (Uganda Virus Research Institute Research Ethics Committee), May 2019 (National Drug Authority and Uganda National Council for Science and Technology) and June 2019 (London School of Hygiene and Tropical Medicine). Collaborator/investigator/trial steering committee meetings were also held during the initial 12-month planning period. Recruitment is scheduled to commence in May 2020. Intervention will be up to 12 months, with completion of the project scheduled for April 2022.

For peer review only

For peer review only - http://bmjopen.bmj.com/site/about/guidelines.xhtml

Competing interests

Alison Elliott reports a grant from the Medical research Council, UK (POPVAC programme funding). The rest of the authors declare that they have no conflicts of interest.

Author contributions

AME conceived the study. AME, GN, ELW, AN, AW, SC, LZ and MM contributed to study design. LZ, GO, GK, JS, CO, MN, EN, FA and JT are site clinicians/nurses/clinical laboratory technicians providing valuable input on clinical considerations of the intervention. MS, SK, FK, RK and MK are field workers and administrators handling the organisational integration of the intervention. AN, AM, HA and ELW are involved in organisation of the databases, trial randomization, treatment allocation and drawing up of analytical plans. LZ, GN, JN, AN, SC, ELW and AME drafted the manuscript. All authors reviewed the manuscript, contributed to it and approved the final version.

Acknowledgements

We thank the Uganda National Expanded Programme for Immunisation, Sanofi Pasteur and PaxVax for providing the HPV, yellow fever and oral typhoid vaccines, respectively. The BCG and tetanus-diphtheria vaccines were kind donations from the Serum Institute of India. We thank the Wakiso district local government and Entebbe hospital for their support. We also thank members of the POPVAC programme steering committee (chaired by Prof. Richard Hayes) and the Data and Safety Monitoring Board (Dr David Meya, Prof. Andrew Prendergast and Dr Elizabeth George).

Funding

The POPVAC programme of work is supported by the Medical Research Council of the United Kingdom (MR/R02118X/1). SC and JN were supported in part by the Makerere University – Uganda Virus Research Institute Centre of Excellence for Infection and Immunity Research and Training (MUII-plus). MUII-plus is funded under the DELTAS Africa Initiative. The DELTAS Africa Initiative is an independent funding scheme of the African Academy of Sciences (AAS), Alliance for Accelerating Excellence in Science in Africa (AESA) and supported by the New Partnership for Africa's Development Planning and Coordinating Agency (NEPAD Agency) with funding from the Wellcome Trust (grant 107743) and the UK Government. The MRC/UVRI and LSHTM Uganda Research Unit is jointly funded by the UK Medical Research Council (MRC) and the UK Department for International Development (DFID) under the MRC/DFID Concordat agreement and is also part of the EDCTP2 programme supported by the European Union.

For peer review only - http://bmjopen.bmj.com/site/about/guidelines.xhtml

The study sponsor (London School of Hygiene and Tropical Medicine) and funders had no role in study design; collection, management, analysis, and interpretation of data; writing of the protocol; and the decision to submit the protocol for publication.

POPVAC trial team

Principal investigator**:* Alison Elliott; ***Project leader: Ludoviko Zirimenya; ***laboratory staff**:* Gyaviira Nkurunungi, Stephen Cose, Rebecca Amongin, Beatrice Nassanga, Jacent Nassuuna, Irene Nambuya, Prossy Kabuubi, Emmanuel Niwagaba, Gloria Oduru, Grace Kabami; ***statisticians and data managers**:* Emily Webb, Agnes Natukunda, Helen Akurut, Alex Mutebe; ***clinicians**:* Anne Wajja, Milly Namutebi, Christopher Zziwa, Joel Serubanja; ***nurses**:* Caroline Onen, Esther Nakazibwe, Josephine Tumusiime, Caroline Ninsiima, Susan Amongi, Florence Akello; ***internal monitor**:* Mirriam Akello; ***field workers**:* Robert Kizindo, Moses Sewankambo, Denis Nsubuga, Samuel Kiwanuka, Fred Kiwudhu; ***boatman**:* David Abiriga; ***administrative management**:* Moses Kizza, Samsi Nansukusa; ***internal and external collaborators:*** Pontiano Kaleebu, Hermelijn Smits, Maria Yazdanbakhsh, Govert van Dam, Paul Corstjens, Sarah Staedke, Henry Luzze, James Kaweesa, Edridah Tukahebwa, Elly Tumushabe, Moses Muwanga.

For peer review only

REFERENCES

1. Benn CS, Netea MG, Selin LK, et al. A small jab - a big effect: nonspecific immunomodulation by vaccines. *Trends in immunology* 2013;34(9):431-9. doi: 10.1016/j.it.2013.04.004 [published Online First: 2013/05/18]

2. Rieckmann A, Villumsen M, Sorup S, et al. Vaccinations against smallpox and tuberculosis are associated with better long-term survival: a Danish case-cohort study 1971-2010. *International journal of epidemiology* 2017;46(2):695-705. doi: 10.1093/ije/dyw120 [published Online First: 2016/07/07]

3. Prentice S, Webb EL, Dockrell HM, et al. Investigating the non-specific effects of BCG vaccination on the innate immune system in Ugandan neonates: study protocol for a randomised controlled trial. *Trials* 2015;16:149. doi: 10.1186/s13063-015-0682-5

4. Biering-Sørensen S, Aaby P, Lund N, et al. Early BCG-Denmark and Neonatal Mortality Among Infants Weighing <2500 g: A Randomized Controlled Trial. *Clinical infectious diseases : an official publication of the Infectious Diseases Society of America* 2017;65(7):1183-90. doi: 10.1093/cid/cix525 [published Online First: 2018/03/27]

5. Blok BA, Arts RJ, van Crevel R, et al. Trained innate immunity as underlying mechanism for the long-term, nonspecific effects of vaccines. *J Leukoc Biol* 2015;98(3):347-56. doi: 10.1189/jlb.5RI0315-096R

6. Kleinnijenhuis J, Quintin J, Preijers F, et al. Bacille Calmette-Guerin induces NOD2-dependent nonspecific protection from reinfection via epigenetic reprogramming of monocytes. *Proceedings of the National Academy of Sciences of the United States of America* 2012;109(43):17537-42. doi: 10.1073/pnas.1202870109

7. de Bree LCJ, Koeken V, Joosten LAB, et al. Non-specific effects of vaccines: Current evidence and potential implications. *Semin Immunol* 2018;39:35-43. doi: 10.1016/j.smim.2018.06.002 [published Online First: 2018/07/17]

8. van der Heijden C, Noz MP, Joosten LAB, et al. Epigenetics and Trained Immunity. *Antioxid Redox Signal* 2018;29(11):1023-40. doi: 10.1089/ars.2017.7310 [published Online First: 2017/10/06]

9. Rusek P, Wala M, Druszczynska M, et al. Infectious Agents as Stimuli of Trained Innate Immunity. *International journal of molecular sciences* 2018;19(2) doi: 10.3390/ijms19020456 [published Online First: 2018/02/07]

10. Schrum JE, Crabtree JN, Dobbs KR, et al. Cutting Edge: Plasmodium falciparum Induces Trained Innate Immunity. *Journal of immunology (Baltimore, Md : 1950)* 2018;200(4):1243-48. doi: 10.4049/jimmunol.1701010 [published Online First: 2018/01/14]

11. Dayal D, Gupta S. Connecting BCG Vaccination and COVID-19: Additional Data. *medRxiv* 2020:2020.04.07.20053272. doi: 10.1101/2020.04.07.20053272

12. Miller A, Reandelar MJ, Fasciglione K, et al. Correlation between universal BCG vaccination policy and reduced morbidity and mortality for COVID-19: an epidemiological study. *medRxiv* 2020:2020.03.24.20042937. doi: 10.1101/2020.03.24.20042937

13. Leentjens J, Kox M, Stokman R, et al. BCG Vaccination Enhances the Immunogenicity of Subsequent Influenza Vaccination in Healthy Volunteers: A Randomized, Placebo-Controlled Pilot Study. *The Journal of infectious diseases* 2015;212(12):1930-8. doi: 10.1093/infdis/jiv332

14. Arts RJW, Moorlag S, Novakovic B, et al. BCG Vaccination Protects against Experimental Viral Infection in Humans through the Induction of Cytokines Associated with Trained Immunity. *Cell host & microbe* 2018;23(1):89-100.e5. doi: 10.1016/j.chom.2017.12.010 [published Online First: 2018/01/13]

15. Webb EL, Mawa PA, Ndibazza J, et al. Effect of single-dose anthelmintic treatment during pregnancy on an infant's response to immunisation and on susceptibility to infectious diseases in infancy: a randomised,

For peer review only - http://bmjopen.bmj.com/site/about/guidelines.xhtml

double-blind, placebo-controlled trial. *Lancet* 2011;377(9759):52-62. doi: 10.1016/S0140-6736(10)61457-2 [published Online First: 2010/12/24]

16. Barreto ML, Pilger D, Pereira SM, et al. Causes of variation in BCG vaccine efficacy: examining evidence from the BCG REVAC cluster randomized trial to explore the masking and the blocking hypotheses. *Vaccine* 2014;32(30):3759-64. doi: 10.1016/j.vaccine.2014.05.042

17. Chan AW, Tetzlaff JM, Altman DG, et al. SPIRIT 2013 statement: defining standard protocol items for clinical trials. *Ann Intern Med* 2013;158(3):200-7. doi: 10.7326/0003-4819-158-3-201302050-00583 [published Online First: 2013/01/09]

18. Harris PA, Taylor R, Minor BL, et al. The REDCap Consortium: Building an International Community of Software Platform Partners. *Journal of biomedical informatics* 2019:103208. doi: 10.1016/j.jbi.2019.103208 [published Online First: 2019/05/13]

19. Harris PA, Taylor R, Thielke R, et al. Research electronic data capture (REDCap)--a metadata-driven methodology and workflow process for providing translational research informatics support. *Journal of biomedical informatics* 2009;42(2):377-81. doi: 10.1016/j.jbi.2008.08.010 [published Online First: 2008/10/22]

20. Muyanja E, Ssemaganda A, Ngauv P, et al. Immune activation alters cellular and humoral responses to yellow fever 17D vaccine. *The Journal of clinical investigation* 2014;124(7):3147-58. doi: 10.1172/jci75429 [published Online First: 2014/06/10]

21. Akondy RS, Johnson PL, Nakaya HI, et al. Initial viral load determines the magnitude of the human CD8 T cell response to yellow fever vaccination. *Proceedings of the National Academy of Sciences of the United States of America* 2015;112(10):3050-5. doi: 10.1073/pnas.1500475112 [published Online First: 2015/02/26]

22. Sabin EA, Araujo MI, Carvalho EM, et al. Impairment of tetanus toxoid-specific Th1-like immune responses in humans infected with Schistosoma mansoni. *The Journal of infectious diseases* 1996;173(1):269-72. [published Online First: 1996/01/01]

23. Elliott AM, Mawa PA, Webb EL, et al. Effects of maternal and infant co-infections, and of maternal immunisation, on the infant response to BCG and tetanus immunisation. *Vaccine* 2010;29(2):247-55. doi: 10.1016/j.vaccine.2010.10.047

24. Brown J, Baisley K, Kavishe B, et al. Impact of malaria and helminth infections on immunogenicity of the human papillomavirus-16/18 AS04-adjuvanted vaccine in Tanzania. *Vaccine* 2014;32(5):611-7. doi: 10.1016/j.vaccine.2013.11.061

25. Plotkin SA. Correlates of protection induced by vaccination. *Clinical and vaccine immunology : CVI* 2010;17(7):1055-65. doi: 10.1128/cvi.00131-10 [published Online First: 2010/05/14]

26. Riner DK, Ndombi EM, Carter JM, et al. Schistosoma mansoni Infection Can Jeopardize the Duration of Protective Levels of Antibody Responses to Immunizations against Hepatitis B and Tetanus Toxoid. *PLoS neglected tropical diseases* 2016;10(12):e0005180. doi: 10.1371/journal.pntd.0005180

27. Fletcher HA, Snowden MA, Landry B, et al. T-cell activation is an immune correlate of risk in BCG vaccinated infants. *Nat Commun* 2016;7:11290. doi: 10.1038/ncomms11290

28. Safaeian M, Porras C, Pan Y, et al. Durable antibody responses following one dose of the bivalent human papillomavirus L1 virus-like particle vaccine in the Costa Rica Vaccine Trial. *Cancer prevention research (Philadelphia, Pa)* 2013;6(11):1242-50. doi: 10.1158/1940-6207.capr-13-0203 [published Online First: 2013/11/06]

29. WHO. Immunization, vaccines and biologicals. Vaccine position papers. 2017

For peer review only

For peer review only - http://bmjopen.bmj.com/site/about/guidelines.xhtml

30. Aaby P, Benn C, Nielsen J, et al. Testing the hypothesis that diphtheria–tetanus–pertussis vaccine has negative non-specific and sex-differential effects on child survival in high-mortality countries. *BMJ Open* 2012;2(3):e000707. doi: 10.1136/bmjopen-2011-000707

31. Krishnan A, Srivastava R, Dwivedi P, et al. Non-specific sex-differential effect of DTP vaccination may partially explain the excess girl child mortality in Ballabgarh, India. *Trop Med Int Health* 2013;18(11):1329-37. doi: 10.1111/tmi.12192 [published Online First: 2013/10/10]

For peer review only

For peer review only - http://bmjopen.bmj.com/site/about/guidelines.xhtml

SUPPLEMENTARY INFORMATION

The impact of Bacillus Calmette-Guérin revaccination on the response to unrelated vaccines in a Ugandan adolescent birth cohort: randomised controlled trial protocol C for the 'POPulation differences in VACcine responses' (POPVAC) programme

Ludoviko Zirimenya[1,¶], Gyaviira Nkurunungi[1,¶,*], Jacent Nassuuna[1,¶], Agnes Natukunda[1,¶], Alex Mutebe[1], Gloria Oduru[1], Grace Kabami[1], Hellen Akurut[1], Caroline Onen[1], Milly Namutebi[1], Joel Serubanja[1], Esther Nakazibwe[1], Florence Akello[1], Josephine Tumusiime[1], Moses Sewankambo[1], Samuel Kiwanuka[1], Fred Kiwudhu[1], Robert Kizindo[1], Anne Wajja[1], Stephen Cose[1,2], Moses Muwanga[3], Emily L Webb[4], Alison M Elliott[1,2] **for the POPVAC trial team**

[1]Immunomodulation and Vaccines Programme, Medical Research Council/Uganda Virus Research Institute and London School of Hygiene and Tropical Medicine (MRC/UVRI and LSHTM) Uganda Research Unit, Entebbe, Uganda

[2]Department of Clinical Research, London School of Hygiene and Tropical Medicine, London, United Kingdom

[3]Entebbe Hospital, Entebbe, Uganda

[4]MRC Tropical Epidemiology Group, Department of Infectious Disease Epidemiology, London School of Hygiene and Tropical Medicine, London, United Kingdom

¶These authors contributed equally

***Correspondence:** Gyaviira Nkurunungi; Gyaviira.Nkurunungi@mrcuganda.org

For peer review only

Table S1: Schedule of visits and procedures

VISIT NUMBER	1	2	3	3.2, 3.3	4	5	6
WEEKS FROM 1ST IMMUNISATION	-4 to 0[1]	0	4	4 weeks +4 days	8	28	52<br>SE
	Screening	Immunisation	Immunisations		Primary endpoint (PE)	Immunisations	Secondary endpoint (SE)
**RANDOMISED BCG "IMMUNISATION"**							
BCG arm (x)		**x**					
No BCG arm (o)		**o**					
**ANTHELMINTHIC TREATMENT**							
Praziquantel and albendazole or mebendazole					**X**[2]	**X**[2]	**X**[2]
**VACCINES**							
YF-17D			x				
Ty21a			X[6]				
HPV			x		[x][4]	x	
Td						x	[x][5]
**INVESTIGATIONS/PROCEDURES**							
Inclusion/exclusion criteria	x						
Informed consent	x						
Questionnaire	x		x	x	x	x	x
Examination	x		(x)	(x)	(x)	(x)	(x)
Urine β-HCG test (female only) 1mL	x	X[5]	x			x	
Urine YF viral load				x			
Stool for PCR and storage	x						x
Stool for coproantibody and storage	x				x		
**BLOOD TESTS**							
Malaria PCR (1ml)	x						x
Serology for HIV, prior malaria and *S. mansoni* (0.5 ml)	x						
Mansonella perstans (1ml)	x						
Full blood count (1ml)	x		x				
Assessments of pre-immunisation responses, and/or vaccine response outcomes and/or exploratory immunology; storage[9] (10-20ml)	x		x		x		x
Blood for gene expression (2ml)	x		x				
Blood vol (mL)	27		17		20		25
Cumulative blood vol (mL)[8]	27		44		64		89

PE: primary endpoint; **SE:** secondary endpoint
Immunisation days highlighted in green, primary end point days in red
(**x**) performed if clinically indicated
1. Screening and enrolment into Project C will take place shortly before enrolment, sometimes on the same day

For peer review only - http://bmjopen.bmj.com/site/about/guidelines.xhtml

2. Treatments given after sample when schedules coincide
3. Week 8 HPV dose will be given for previously-unvaccinated girls aged ≥14 years
4. Week 52 Td booster dose will be provided as a service
5. Pregnancy test to be repeated if more than 4 weeks elapses between screening and immunisation
6. Oral typhoid vaccine doses will be administered on three alternate days namely visit 3, 3.1, and 3.2
7. Exploratory immunology blood volume will be guided by guidelines from Harvard Mass General, where a maximum of 3ml/kg body weight is taken at any one time point and not more than 3ml/kg is taken over any 8-week period (ref http://www.drgreene.com/21_1616.html.) These guidelines have been followed in a previous study vaccinating adolescents with investigational tuberculosis vaccine MVA85A (in Uganda).[1] The total blood volume planned is 64 ml over the initial intensive sampling period of 8 weeks. Revision of sample volumes based on weight will only be required for participants who weigh less than 21 kg; the average weight of children aged 9 years is expected to be 28kg (with 21kg the 3rd centile) with greater weights for older children.[2]

For peer review only

Further rationale for the selection of vaccines

Yellow fever vaccine

Yellow fever vaccine YF-17D is a live replicating parenteral vaccine. The vaccine (Stamaril; Sanofi Pasteur) is available for purchase in Uganda. Yellow Fever (YF) causes outbreaks in Uganda and the wider region[3] and YF-17D is a candidate for Uganda's expanded programme on immunisation (EPI; H Luzze, personal communication). As noted above, lower vaccine replication, lower neutralising antibody induction, and greater waning, are described in Uganda compared to Switzerland.[4] YF-17D is a potential vector for novel vaccine constructs,[5] adding relevance to vaccine development.

Typhoid vaccine Ty21a

Typhoid vaccine Ty21a is a live replicating oral vaccine and also a potential vector for new vaccine constructs.[6] Ty21a vaccine will be purchased from PaxVax, Redwood City, California. Substantial, multi-year typhoid outbreaks occur in Uganda and immunisation campaigns have been advocated as cost effective.[7]

Ty21a was developed in the 1970s. Although not routinely used in Uganda, it has been (and is currently) registered in many countries. It was first registered in the United States and United Kingdom in the 1980s, and is recommended by the World Health Organisation for both endemic and epidemic settings.[8] It has comparable efficacy to the parenteral Vi polysaccharide typhoid vaccine, good durability and minimal adverse effects.[8] It is proposed for use in this study to model effects of study exposures and intervention on the response to a live oral vaccine.

The Ty21a vaccine is given as a three-dose regimen on alternate days.

Human Papilloma Virus (HPV) vaccine

Human Papilloma Virus (HPV) vaccine is a protein virus-like particle. The quadrivalent HPV Vaccine Gardasil (Merck) is available for purchase in Uganda and is the vaccine used by the national EPI programme. HPV immunisation is being rolled out among girls to prevent cervical neoplasia, the commonest cancer among Ugandan women and we will coordinate provision with the national HPV immunisation programme.[9] HPV immunisation is also beneficial for boys since HPV infection is associated with anogenital warts, anal cancer and oropharyngeal cancers in both males and females, and with penile cancer in men,[10] and we will include boys in these studies.

Tetanus and diphtheria vaccines

Tetanus and diphtheria vaccines comprise inert toxoids (Td). Booster immunisation is recommended for young women to prevent maternal and neonatal tetanus. Recent evidence emphasises the need to protect young men also.[11]

For peer review only - http://bmjopen.bmj.com/site/about/guidelines.xhtml

For peer review only

Immunisation Postponement Criteria

If any one of the following is identified at the time scheduled for immunisation, the participant may be immunised at a later date, or withdrawn, at the discretion of the Investigator. The participant must be followed until resolution of the event as with any adverse event:

- Acute disease at the time of immunisation. Acute disease is defined as the presence of a moderate or severe illness with or without fever. All vaccines can be administered to persons with a minor illness such as diarrhoea or mild upper respiratory infection with or without low-grade fever, i.e. temperature of ≤37.5°C (99.5°F)
- Temperature of >37.5°C (99.5°F) at the time of immunisation
- Taking antibiotics or antimalarials currently, or within the past 7 days, of the date of Ty21a administration (ascertained verbally)

Vaccine storage and transport

In order to maintain a reliable vaccine cold chain, the vaccines and diluents to be used will be stored and transported within the recommended temperature range of +2ºC to +8ºC. Care will be taken to ensure that the vaccines are not frozen. BCG, being sensitive to light, will be kept in the dark (normally within its secondary packaging) for as long as possible to protect it during storage and transportation. All vaccines will be kept in appropriate refrigeration equipment with a temperature monitoring device to ensure temperatures remain between +2ºC and +8ºC. Cold boxes/vaccines carriers with temperature monitors will be used to transport vaccines and the diluents from the MRC/UVRI and LSHTM Uganda Research Unit (Entebbe) to the clinic where vaccination will take place and while transporting vaccines to immunisation sessions. Designated staff will be given responsibility for managing the vaccine cold chain. All cold chain equipment including the temperature monitoring devices used for this project will comply with relevant technical specifications as defined by the EPI standards. Basic routine maintenance will be regularly carried out on all cold chain equipment.

Additional laboratory measurements

Additional assays will comprise measurement of parasite infection exposure, HIV serology, pregnancy testing and full blood counts. HIV testing and pregnancy testing will be accompanied by appropriate counselling by trained staff.

Current *S. mansoni* infection status and intensity will be determined by serum/plasma levels of circulating anodic antigen (CAA). The method is quantitative, highly specific for *Schistosoma* infection, and much more sensitive than the conventional Kato Katz method.[12] CAA will be assessed retrospectively on stored samples collected at baseline.

For peer review only

Prior exposure to schistosomiasis will be evaluated by ELISA for IgG to schistosome egg antigen using stored blood samples collected at baseline.

The presence of other helminth infections will be determined retrospectively using stool PCR of samples collected at baseline and at weeks 28 and 52.[13] In accordance with national guidelines, all participants will be treated with albendazole or mebendazole after collection of samples for primary endpoints at week 8 and 28, and after collection of samples for secondary endpoints at week 52.

Current malaria infection status and intensity will be assessed retrospectively by PCR on stored samples collected on immunisation days and at week 52.

Malarial fever: Individuals presenting with fever will be investigated using rapid diagnostic tests for malaria and treated based on the results and according to prevailing national guidelines.

Prior malaria exposure will be evaluated by ELISA for IgG to malaria antigen using stored samples collected at baseline.

HIV serology will be done on blood samples using rapid tests and according to prevailing national algorithms. The current algorithm is shown in Appendix 2. This will be done at baseline.

Pregnancy testing will be done using urine samples and standard operating procedures for assessment of urine [illegible]-human chorionic gonadotropin[illegible]hCG). This will be done at baseline and before immunisation on each immunisation day.

Full blood counts will be conducted using a haematology analyser. Mild, moderate and severe anaemia will be defined according to WHO guidelines, by age.[14] This will be done at baseline (to test for anaemia as part of the eligibility assessment), and pre-immunisation as part of the assessment of immunological profile.

Individuals found to be HIV positive or pregnant will be referred to appropriate providers for further care.

Individuals with severe anaemia (haemoglobin <82g/L) will be excluded from the randomised intervention (since the intervention might be beneficial in management of anaemia). They will be treated for anaemia and for any underlying cause identified.

Operational considerations

Programme governance

A Programme Steering Committee will be set up to guide progress across all projects. This will comprise the following:

For peer review only

For peer review only - http://bmjopen.bmj.com/site/about/guidelines.xhtml

118 • An independent chair
119 • Representatives from the Ministry of Health programmes for immunisation and for vector borne disease control
121 • Representatives of district authorities (Mukono and Jinja districts)
122 • Community representatives
123 • Principal investigator and co-investigators
124 • Project leader and post-doctoral immunologist
125 • Trial statistician
126 • Laboratory manager
127 • Medical Research Council observer

128 *Informed consent*

129 Both written informed assent from the participants and written informed consent from a parent or guardian will be required for participation, although these may not necessarily be obtained at the same time. Information will be provided in both English and the appropriate local language. For individuals who cannot speak the languages used, or who cannot read or write, a witness who can read the information sheet and translate the information to the participant or parent/guardian will be used. Informed consent by emancipated or mature minors will be obtained using designated consent form for these kinds of participants.

136 The aims of the study, all tests, treatments and immunisations to be carried out and potential risks will be explained. The participant will be given the opportunity to ask about details of the trial, and will then have time to consider whether or not to participate. If they do decide to participate, they and their parent/guardian will sign and date two copies of the assent and consent forms, one for them to take away and keep, and one to be stored securely by the research team. Separate information and consent forms will be provided for consent for storage of samples for future studies and for anonymous sharing of data from this study. For the EMaBS cohort genetic data are already available based on previous approval; the information sheet will explain that these data may be used in analyses related to this protocol.

145 *Screening and Eligibility Assessment*

146 Once the informed consent process has been completed, and consent (and assent) given, a baseline medical history (including concomitant medication) will be collected. Vital signs will be checked and a physical examination will be performed. Inclusion and exclusion criteria will be checked.

For peer review only - http://bmjopen.bmj.com/site/about/guidelines.xhtml

Participants will undergo pre- and post-test counselling for HIV and (for girls) pregnancy testing by a trained and experienced nurse- or clinician-counsellor. Blood, urine and stool samples will be obtained, for tests as specified in the schedule of procedures (Appendices A-C). These tests are to exclude the major, immunomodulating co-infection, HIV, and conditions that might impact safety (anaemia, pregnancy).

Enrolment

Participants who consent/assent, complete the screening processes, satisfy all the inclusion criteria and meet none of the exclusion criteria will be enrolled into the trial. On the enrolment day (which may be the same as the screening day in some cases) eligibility will be checked and participants will be enrolled sequentially to the next randomisation number. They will then be given BCG vaccine or not, according to their allocation.

Discontinuation / withdrawal criteria

In accordance with the principles of the current revision of the Declaration of Helsinki and any other applicable regulations, a participant has the right to withdraw from the study at any time and for any reason, and is not obliged to give his or her reasons for doing so. The Investigator may withdraw the participant at any time in the interests of the participant's health and well-being. In addition, the participant may withdraw/be withdrawn for any of the following reasons:

- Ineligibility (either arising during the study or retrospectively, having been overlooked at screening)
- Administrative decision by the Investigator
- Significant protocol deviation
- Participant non-compliance with study requirements
- An adverse event which requires discontinuation of the study involvement or results in inability to continue to comply with study procedures.

Any participant who becomes pregnant during the trial will be followed up until the end of the pregnancy but no further immunisations will be given unless indicated during pregnancy (as is the case for tetanus toxoid). The trial allocation for this participant will be unblinded and the participant will only be given further treatment if clinically indicated. The babies will also be followed up and examined for any adverse effects. We will not routinely perform venepuncture in a pregnant participant.

For peer review only

For peer review only - http://bmjopen.bmj.com/site/about/guidelines.xhtml

The reason for withdrawal will be recorded in the case report form (CRF). If withdrawal is due to an AE, appropriate follow-up visits or medical care will be arranged, with the agreement of the participant, until the AE has resolved, stabilised or a non-trial related causality has been assigned.

If a participant withdraws from the study samples collected before their withdrawal from the trial will be used/ stored unless the participant specifically requests otherwise.

Trial discontinuation

The Trial will be discontinued in the event of new scientific information that renders continuation futile or unethical, or for any other reason, at the discretion of the Programme Steering Committee.

End of study definition

The trial will be completed when the last participant enrolled into the trial has completed their final follow up visit.

Safety assessments and oversight

No new investigational drug or product will be used in the proposed trial. However, standard approaches for monitoring safety and reporting of serious adverse events will be followed.

Monitoring

The trial will be monitored by both internal and external monitors according to a pre-defined monitoring plan which will include a site initiation visit, monitoring visits at least annually, and a close-out visit. The monitors will assess patient safety, data integrity, and adherence to the protocol and to Good Clinical Research Practice procedures.

Procedures to be followed in the event of abnormal findings

Abnormal clinical findings from medical history, examination or blood tests will be assessed as to their clinical significance throughout the trials. If an abnormal test result is deemed clinically significant, it may be repeated. If a test remains clinically significant, the participant will be informed and appropriate medical care arranged as appropriate and with the permission of the participant. Specific details regarding findings, discussion with participants and resulting actions will be recorded in the clinical records. Decisions to exclude the participant from enrolling in the trial or to withdraw a participant from the trial will be at the discretion of the Investigator.

For peer review only

For peer review only - http://bmjopen.bmj.com/site/about/guidelines.xhtml

Data and Safety Monitoring Board (DSMB)

A data and safety monitoring board (DSMB) will be appointed to provide real-time safety oversight. The DSMB will be notified within 7 days of the Investigators' being aware of the occurrence of SAEs. The DSMB may recommend the Investigators to place the trial on hold if deemed necessary following an intervention-related SAE. The DSMB will be chaired by a clinician experienced in clinical trials. There will be a minimum of two other appropriately qualified committee members. In the case of events related to a blinded intervention, the DSMB can request unblinding. Membership will include a statistician, and at least one Ugandan member. All correspondence between Investigators and the DSMB will be conveyed by the Principal Investigator to the trial Sponsor. The Chair of the DSMB will be contacted for advice and independent review by the Investigator or trial Sponsor in the following situations:

- The occurrence of any SAE
- Any other situation where the Investigator or trial Sponsor feels independent advice or review is important.

Ethical and regulatory considerations

Information regarding risks and benefits to the participant

Participants in this programme will be adolescents and therefore a vulnerable human population. Care will be taken to provide adequate, age and education-status appropriate information and to ensure that it is understood; and to emphasise that participation is voluntary. Participants will be enrolled only when they have given their own assent and when consent has been given by the parent or guardian.

No major risks to the participants are anticipated since all the treatments and vaccines to be given are licensed and known to be safe. The main risk to participants will be time lost from school work: we will work with parents to minimise disruption to studies.

Participants will suffer the discomfort and inconvenience of providing blood samples (and stool and urine samples). Occasionally people faint when a vaccine is given or when blood is drawn. Individuals will be comfortably seated during these procedures and the research team will be trained to manage such events.

The immunisations to be given have recognised side effects which are usually mild and resolve spontaneously in a few days to one week. Parenteral vaccines are likely to result in pain and swelling at the site of injection and mild fever; very occasionally pain and swelling can be severe and associated with difficulty in moving the shoulder. Sometimes headache and tiredness occurs. Rarely

For peer review only

a vaccine may cause a severe allergic reaction. For most vaccines this is estimated at less than one in a million doses (but 1 in 55,000 for Yellow Fever vaccine).[15] Individuals with a history of a possible allergic reaction to drugs or vaccines, or to vaccine components including eggs or chicken proteins, will be excluded from the studies. The research team will be trained and prepared to manage severe allergic reactions.

Adverse reactions to Yellow Fever vaccine include severe nervous system reaction (about 1 person in 125,000) and severe, life-threatening illness with organ failure (about 1 person in 250,000). The mortality for this severe, life-threatening adverse effect is reported as about 50%.[15]

BCG immunisation is likely to induce a scar in many cases. This may develop over several weeks, starting as a small papule at the injection site which may become ulcerated and then heal over a period of 2 to 5 months; and lymphadenopathy may develop. Occasionally a more severe local reaction occurs (estimated at 1 per 1,000-10,000 doses): for example, an abscess develops and scars may develop into keloids. Rarely BCG can cause disseminated disease (1 per 230,000 to 640,000 doses), or disease in sites remote from the immunisation site. Disseminated BCG disease usually occurs in immunocompromised people: HIV positive people will be excluded from these studies.[16] BCG "pre-immunisation" may interfere with the response to the subsequent live vaccines; indeed our hypothesis, and published results, suggest that it may suppress replication of YF 17D vaccine.[17] However, this reduced replication has not been shown to correlate with, or result in, reduced levels of neutralising antibody titres (which are the desired protective outcome).[4,17]

Oral typhoid vaccine (Ty21a) may occasionally be associated with stomach pain, nausea, vomiting and (rarely) rash.[15]

Benefits

All the vaccines to be given are licensed and regarded as safe. In general, the vaccines and treatments are expected to provide protection against infectious diseases. Participants and their families, and communities are expected to benefit from improved understanding of vaccines.

For peer review only

For peer review only - http://bmjopen.bmj.com/site/about/guidelines.xhtml

References

1. Wajja A, Kizito D, Nassanga B, et al. The effect of current Schistosoma mansoni infection on the immunogenicity of a candidate TB vaccine, MVA85A, in BCG-vaccinated adolescents: An open-label trial. *PLoS Negl Trop Dis* 2017;11(5):e0005440. doi: 10.1371/journal.pntd.0005440 [published Online First: 2017/05/05]

2. WHO. Growth reference 5-19 years. 2007

3. Kraemer MU, Faria NR, Reiner RC, Jr., et al. Spread of yellow fever virus outbreak in Angola and the Democratic Republic of the Congo 2015-16: a modelling study. *The Lancet Infectious diseases* 2016 doi: 10.1016/s1473-3099(16)30513-8 [published Online First: 2016/12/27]

4. Muyanja E, Ssemaganda A, Ngauv P, et al. Immune activation alters cellular and humoral responses to yellow fever 17D vaccine. *The Journal of clinical investigation* 2014;124(7):3147-58. doi: 10.1172/jci75429 [published Online First: 2014/06/10]

5. Aguiar M, Stollenwerk N, Halstead SB. The Impact of the Newly Licensed Dengue Vaccine in Endemic Countries. *PLoS neglected tropical diseases* 2016;10(12):e0005179. doi: 10.1371/journal.pntd.0005179 [published Online First: 2016/12/22]

6. Dharmasena MN, Osorio M, Filipova S, et al. Stable expression of Shigella dysenteriae serotype 1 O-antigen genes integrated into the chromosome of live Salmonella oral vaccine vector Ty21a. *Pathogens and disease* 2016 doi: 10.1093/femspd/ftw098 [published Online First: 2016/09/23]

7. Carias C, Walters MS, Wefula E, et al. Economic evaluation of typhoid vaccination in a prolonged typhoid outbreak setting: the case of Kasese district in Uganda. *Vaccine* 2015;33(17):2079-85. doi: 10.1016/j.vaccine.2015.02.027 [published Online First: 2015/02/26]

8. WHO. Position Paper on Typhoid vaccines: WHO position paper – March 2018 2018

9. Centre HI. HPV and related diseases report: Uganda. 2016. http://www.hpvcentre.net/statistics/reports/UGA.pdf (accessed 20.01.2017).

10. WHO. Human papillomavirus vaccines: WHO position paper, May 2017. *Releve epidemiologique hebdomadaire* 2017;92(19):241-68. [published Online First: 2017/05/23]

11. Nanteza B, Galukande M, Aceng J, et al. The burden of tetanus in Uganda. *SpringerPlus* 2016;5(1):705. doi: 10.1186/s40064-016-2309-z [published Online First: 2016/06/29]

12. Corstjens PL, Nyakundi RK, de Dood CJ, et al. Improved sensitivity of the urine CAA lateral-flow assay for diagnosing active Schistosoma infections by using larger sample volumes. *Parasites & vectors* 2015;8:241. doi: 10.1186/s13071-015-0857-7 [published Online First: 2015/04/22]

13. Webb EL, Nampijja M, Kaweesa J, et al. Helminths are positively associated with atopy and wheeze in Ugandan fishing communities: results from a cross-sectional survey. *Allergy* 2016 doi: 10.1111/all.12867

14. WHO. Haemoglobin concentrations for the diagnosis of anaemia and assessment of severity. Vitamin and Mineral Nutrition Information System. Geneva, World Health Organization, 2011 (WHO/NMH/NHD/MNM/11.1).

15. CDC. Centers for Disease Control and Prevention, vaccines and immunizations.

16. WHO. Information sheet observed rate of vaccine reactions Bacille Calmette-Guérin (BCG) vaccine. 2012

For peer review only

301 17. Arts RJW, Moorlag S, Novakovic B, et al. BCG Vaccination Protects against Experimental Viral Infection in Humans through the Induction of Cytokines Associated with Trained Immunity. *Cell host & microbe* 2018;23(1):89-100.e5. doi: 10.1016/j.chom.2017.12.010 [published Online First: 2018/01/13]

For peer review only

For peer review only - http://bmjopen.bmj.com/site/about/guidelines.xhtml

Reporting checklist for protocol of a clinical trial.

Based on the SPIRIT guidelines.

Instructions to authors

Complete this checklist by entering the page numbers from your manuscript where readers will find each of the items listed below.

Your article may not currently address all the items on the checklist. Please modify your text to include the missing information. If you are certain that an item does not apply, please write "n/a" and provide a short explanation.

Upload your completed checklist as an extra file when you submit to a journal.

In your methods section, say that you used the SPIRITreporting guidelines, and cite them as:

Chan A-W, Tetzlaff JM, Altman DG, Laupacis A, Gøtzsche PC, Krleža-Jerić K, Hróbjartsson A, Mann H, Dickersin K, Berlin J, Doré C, Parulekar W, Summerskill W, Groves T, Schulz K, Sox H, Rockhold FW, Rennie D, Moher D. SPIRIT 2013 Statement: Defining standard protocol items for clinical trials. Ann Intern Med. 2013;158(3):200-207

For peer review only

		Reporting Item	Page Number
**Administrative information**			
Title	#1	Descriptive title identifying the study design, population, interventions, and, if applicable, trial acronym	1
Trial registration	#2a	Trial identifier and registry name. If not yet registered, name of intended registry	2
Trial registration: data set	#2b	All items from the World Health Organization Trial Registration Data Set	n/a
Protocol version	#3	Date and version identifier	Information available at ISRCTN10482904
Funding	#4	Sources and types of financial, material, and other support	16

Roles and responsibilities: contributorship	#5a	Names, affiliations, and roles of protocol contributors	16
Roles and responsibilities: sponsor contact information	#5b	Name and contact information for the trial sponsor	Information available at ISRCTN10482904
Roles and responsibilities: sponsor and funder	#5c	Role of study sponsor and funders, if any, in study design; collection, management, analysis, and interpretation of data; writing of the report; and the decision to submit the report for publication, including whether they will have ultimate authority over any of these activities	17
Roles and responsibilities: committees	#5d	Composition, roles, and responsibilities of the coordinating centre, steering committee, endpoint adjudication committee, data management team, and other individuals or groups overseeing the trial, if applicable (see Item 21a for data monitoring committee)	Supplementary information – Pg. 6 and 10
**Introduction**			
Background and rationale	#6a	Description of research question and justification for undertaking the trial, including summary of relevant studies (published and unpublished) examining benefits and harms for each intervention	4 and 5
Background and rationale: choice of comparators	#6b	Explanation for choice of comparators	7
Objectives	#7	Specific objectives or hypotheses	6
Trial design	#8	Description of trial design including type of trial (eg, parallel group, crossover, factorial, single group), allocation ratio, and framework (eg, superiority, equivalence, non-inferiority, exploratory)	7

For peer review only

**Methods: Participants, interventions, and outcomes**			
Study setting	#9	Description of study settings (eg, community clinic, academic hospital) and list of countries where data will be collected. Reference to where list of study sites can be obtained	6
Eligibility criteria	#10	Inclusion and exclusion criteria for participants. If applicable, eligibility criteria for study centres and individuals who will perform the interventions (eg, surgeons, psychotherapists)	6 and 7
Interventions: description	#11a	Interventions for each group with sufficient detail to allow replication, including how and when they will be administered	8
Interventions: modifications	#11b	Criteria for discontinuing or modifying allocated interventions for a given trial participant (eg, drug dose change in response to harms, participant request, or improving / worsening disease)	Supplementary information
Interventions: adherance	#11c	Strategies to improve adherence to intervention protocols, and any procedures for monitoring adherence (eg, drug tablet return; laboratory tests)	Supplementary information
Interventions: concomitant care	#11d	Relevant concomitant care and interventions that are permitted or prohibited during the trial	n/a; participants are not expected to be receiving any concomitant care and interventions during the study
Outcomes	#12	Primary, secondary, and other outcomes, including the specific measurement variable (eg, systolic blood pressure), analysis metric (eg, change from baseline, final value, time to event), method of aggregation (eg, median, proportion), and time point for each outcome. Explanation of the clinical relevance of chosen efficacy and harm outcomes is strongly recommended	10

For peer review only

Participant timeline	#13	Time schedule of enrolment, interventions (including any run-ins and washouts), assessments, and visits for participants. A schematic diagram is highly recommended (see Figure)	Supplementary information; Table S1, pg 2
Sample size	#14	Estimated number of participants needed to achieve study objectives and how it was determined, including clinical and statistical assumptions supporting any sample size calculations	11
Recruitment	#15	Strategies for achieving adequate participant enrolment to reach target sample size	12
**Methods: Assignment of interventions (for controlled trials)**			
Allocation: sequence generation	#16a	Method of generating the allocation sequence (eg, computer-generated random numbers), and list of any factors for stratification. To reduce predictability of a random sequence, details of any planned restriction (eg, blocking) should be provided in a separate document that is unavailable to those who enrol participants or assign interventions	8
Allocation concealment mechanism	#16b	Mechanism of implementing the allocation sequence (eg, central telephone; sequentially numbered, opaque, sealed envelopes), describing any steps to conceal the sequence until interventions are assigned	8
Allocation: implementation	#16c	Who will generate the allocation sequence, who will enrol participants, and who will assign participants to interventions	8
Blinding (masking)	#17a	Who will be blinded after assignment to interventions (eg, trial participants, care	8

For peer review only

		providers, outcome assessors, data analysts), and how	
Blinding (masking): emergency unblinding	#17b	If blinded, circumstances under which unblinding is permissible, and procedure for revealing a participant's allocated intervention during the trial	Supplementary information – Pg. 10
**Methods: Data collection, management, and analysis**			
Data collection plan	#18a	Plans for assessment and collection of outcome, baseline, and other trial data, including any related processes to promote data quality (eg, duplicate measurements, training of assessors) and a description of study instruments (eg, questionnaires, laboratory tests) along with their reliability and validity, if known. Reference to where data collection forms can be found, if not in the protocol	13
Data collection plan: retention	#18b	Plans to promote participant retention and complete follow-up, including list of any outcome data to be collected for participants who discontinue or deviate from intervention protocols	13
Data management	#19	Plans for data entry, coding, security, and storage, including any related processes to promote data quality (eg, double data entry; range checks for data values). Reference to where details of data management procedures can be found, if not in the protocol	13.<br><br>These will also be detailed in a statistical analysis plan that will be uploaded to the online trial registration.
Statistics: outcomes	#20a	Statistical methods for analysing primary and secondary outcomes. Reference to where other details of the statistical analysis plan can be found, if not in the protocol	13.<br><br>These will also be detailed in the statistical analysis plan that will be uploaded to the online trial registration.

For peer review only

Statistics: additional analyses	#20b	Methods for any additional analyses (eg, subgroup and adjusted analyses)	13.<br>These will also be detailed in the statistical analysis plan that will be uploaded to the online trial registration.
Statistics: analysis population and missing data	#20c	Definition of analysis population relating to protocol non-adherence (eg, as randomised analysis), and any statistical methods to handle missing data (eg, multiple imputation)	These will be detailed in the statistical analysis plan that will be uploaded to the online trial registration.
**Methods: Monitoring**			
Data monitoring: formal committee	#21a	Composition of data monitoring committee (DMC); summary of its role and reporting structure; statement of whether it is independent from the sponsor and competing interests; and reference to where further details about its charter can be found, if not in the protocol. Alternatively, an explanation of why a DMC is not needed	Supplementary information – Pg. 10
Data monitoring: interim analysis	#21b	Description of any interim analyses and stopping guidelines, including who will have access to these interim results and make the final decision to terminate the trial	Supplementary information – Pg. 9
Harms	#22	Plans for collecting, assessing, reporting, and managing solicited and spontaneously reported adverse events and other unintended effects of trial interventions or trial conduct	Supplementary information – Pg. 10
Auditing	#23	Frequency and procedures for auditing trial conduct, if any, and whether the process will be independent from investigators and the sponsor	Supplementary information – Pg. 9
**Ethics and dissemination**			

For peer review only

Research ethics approval	#24	Plans for seeking research ethics committee / institutional review board (REC / IRB) approval	11
Protocol amendments	#25	Plans for communicating important protocol modifications (eg, changes to eligibility criteria, outcomes, analyses) to relevant parties (eg, investigators, REC / IRBs, trial participants, trial registries, journals, regulators)	11
Consent or assent	#26a	Who will obtain informed consent or assent from potential trial participants or authorised surrogates, and how (see Item 32)	12 and Supplementary information – Pg. 7
Consent or assent: ancillary studies	#26b	Additional consent provisions for collection and use of participant data and biological specimens in ancillary studies, if applicable	Supplementary information – Pg. 7
Confidentiality	#27	How personal information about potential and enrolled participants will be collected, shared, and maintained in order to protect confidentiality before, during, and after the trial	12
Declaration of interests	#28	Financial and other competing interests for principal investigators for the overall trial and each study site	16
Data access	#29	Statement of who will have access to the final trial dataset, and disclosure of contractual agreements that limit such access for investigators	n/a
Ancillary and post trial care	#30	Provisions, if any, for ancillary and post-trial care, and for compensation to those who suffer harm from trial participation	Supplementary information – Pg. 10
Dissemination policy: trial results	#31a	Plans for investigators and sponsor to communicate trial results to participants, healthcare professionals, the public, and other relevant groups (eg, via publication, reporting in results databases, or other data sharing arrangements), including any publication restrictions	2, 11

For peer review only

Dissemination policy: authorship	#31b	Authorship eligibility guidelines and any intended use of professional writers	n/a
Dissemination policy: reproducible research	#31c	Plans, if any, for granting public access to the full protocol, participant-level dataset, and statistical code	11

Appendices

Informed consent materials	#32	Model consent form and other related documentation given to participants and authorised surrogates	Supplementary File
Biological specimens	#33	Plans for collection, laboratory evaluation, and storage of biological specimens for genetic or molecular analysis in the current trial and for future use in ancillary studies, if applicable	n/a

None The SPIRIT checklist is distributed under the terms of the Creative Commons Attribution License CC-BY-ND 3.0. This checklist can be completed online using https://www.goodreports.org/, a tool made by the EQUATOR Network in collaboration with Penelope.ai

For peer review only
